# The relative age effect in young athletes: A countywide analysis of 9–14-year-old participants in all competitive sports

**DOI:** 10.1371/journal.pone.0254687

**Published:** 2021-07-16

**Authors:** Susana M. Gil, Iraia Bidaurrazaga-Letona, Jon Larruskain, Izaro Esain, Jon Irazusta

**Affiliations:** 1 Department of Physiology, Faculty of Medicine and Nursing, University of the Basque Country (UPV/EHU), Leioa, Spain; 2 Medical Services, Athletic Club, Lezama, Spain; Universidade de Evora, PORTUGAL

## Abstract

The relative age effect (RAE) has primarily been investigated in male athletes involved in popular sports and high-level competitions. However, occurrence of RAE in other types of sports at the grassroots level, particularly in female athletes, is less well-studied. Thus, we examined the RAE in a large cohort of young athletes who participated in all competitive sports in Bizkaia, Spain, according to gender and specificity of the sport. The birth dates of 38,381 participants (65.1% males and 34.9% females) aged 9–14 years old in 37 competitive sports were analyzed. Birth dates were divided into four birth-quarters and compared to those of all children born in the same period using a χ^2^ goodness-of-fit test and standardized residuals. The effect size Cramer’s V was measured, and odds ratio and 95% confidence intervals were calculated to determine the odds of athletes born in January playing in the highest leagues. In the total sample, in boys RAE was evident in football, but only in higher-competition leagues (p<0.001, large effect size). In girls, RAE was evident in the most popular team sports: basketball (p<0.001, large effect size in basketball 1^st^ league), handball and football (p<0.05, both small effect sizes). Players born in January were 3.23- and 2.89-times more likely to play in the 1^st^ leagues than those born in December, for boys (football) and girls (basketball) respectively. In the overall analysis and in the remaining sports, presence of RAE was negligible. Therefore, the date of birth does not seem to be a constraint to participating in most sports in Bizkaia. The potential mechanisms for RAE are multifactorial and complex, yet a combination of factors, such as the popularity of a sport and the depth of competition, physicality and social influences may be involved. We discuss these mechanisms and potential measures to mitigate RAE.

## Introduction

To avoid large age differences in sports, children and youth are organized into annual-age groups. These age-specific groups are organized by a selected cut-off date, which in most countries is January 1. Thus, in the same team there may be a member born in January and another member born in December, implying an almost 12-month age difference. A person’s age relative to that of his/her peers within the same annual group is referred to as relative age [1, 2], and its variations are the relative age differences [1, 2].

One of the consequences of these relative age inequalities is the relative age effect (RAE), which describes an overrepresentation of athletes born a few months after the cut-off date (i.e., January, February, and March, when the cut-off date is January 1) as well as underrepresentation of those born at the end of the year [1, 2]. This phenomenon has been demonstrated in a variety of sports [1, 2], mainly in team sports such as soccer [3, 4], ice hockey [5], basketball [6], rugby [7], and handball [8, 9], but also in individual sports such as athletics (track and field) [10, 11], skiing [12], tennis [13, 14], and swimming [15]. Some of these studies included youth [3, 5–7, 10–15] and others senior [4, 6–9, 13] participants. For example, Brustio et al. [16] conducted a large study that included 2064 Italian elite soccer players and observed that 43.3% of players had been born in the first quarter of the year, in contrast to 10.7% born in the last quarter of the year.

The most supported hypothesis explaining the causes for RAE is based on the selection hypothesis [1–16]. This hypothesis suggests that chronologically older children/youth have physical or anthropometrical advantages, which are associated with better performance, and thus are more likely to be identified as more talented and selected into higher-level teams—generating and perpetuating bias toward relatively older players. Likewise, the chances of the relatively younger being selected are thus reduced. The relevance of physicality is confirmed by observation of the RAE particularly in sports where body size and strength are important, as opposed to its nonexistence in weight-categorized sports (taekwondo [17], judo [18], boxing [19] and shooting, in which a large body size is not particularly advantageous [20].

Apart from these physical and physiological advantages, there are also relevant psychological and social influences that may explain RAE in sports [1, 2]. According to Hancock et al. [5], parents may have a relevant role in the genesis of RAE (Mathew effect). One possibility is that parents do not enroll their relatively younger children in sports where physical demands are high [5]. In addition, both athletes’ and coaches’ expectations (Galatea and Pygmalion effects, respectively) toward relatively older athletes may further contribute to this phenomenon [21].

Once players have been included in high-level teams, they have access to high-quality training, equipment, installations, medical staff, etc., thus enhancing their skills consequently, the RAE is established, maintained, and reinforced [1, 2], reaching its highest prevalence at the most prestigious competition levels. Hence, most studies have confirmed the existence of the RAE in the highest-level elite sports, such as World [3, 9, 10] and European Championships [4, 22, 23] and National selections [6].

Further, the RAE indicates that discriminatory practices can deny relatively younger athletes equal participation and prevent their selection for and progress in sports [1, 2]. Undoubtedly, physical activity and sports have many positive influences on child and adolescent health, including essential physical, psychological, and social benefits. According to the literature, it is plausible that children’s birth dates are a key constraining factor to their participation at high levels of competition in sports with a predominantly physical nature [1, 2]. However, whether birth date is a limitation to participating in sports at the lowest grassroots level has not been sufficiently explored. Examination of when selection mechanisms and RAE begin to influence opportunities for participation, development, and performance of children and youth in different sports contexts is warranted [9]. Since it is unclear when the RAE begins, exploring the distribution of birth dates of children and youth engaged in both more popular and less popular sports as well as sports with different features (e.g., individual vs. team, sports organized in weight categories, aesthetic sports) would significantly contribute to understanding of this phenomenon and would provide technical staff and sports authorities with guidance to apply appropriate measures to counterbalance such discrimination, if necessary.

Most studies on RAE have focused on males [1, 3–5, 13–14, 16], and the few studies focused on females have yielded variable results [6, 7, 12, 24, 25]. Regardless, the effect of an uneven distribution of birth dates is more obvious in male than in female athletes [6, 26]. Some authors have failed to demonstrate the presence of RAE in young females [7]. However, increasing evidence for this phenomenon has recently been reported in young females participating in soccer [26], alpine ski racing [12], ice hockey [27], basketball [6], and rugby union [28], as well as in a large cohort of 10–20-year-old participants in tennis, athletics, fencing, and snowboarding [29]. Similarly, both in the London Youth Games study [30] and the Swiss Talent Development Program [31], RAE was present in many of the analyzed sports. In a comprehensive review of the RAE within female sports, Smith et al. [26] concluded that this phenomenon has a small but consistent influence on female sports.

However, most studies are limited to a single sport when analyzing RAE, providing an incomplete picture of the whole phenomenon. We hypothesize that those relatively younger athletes who are not selected to join certain teams try out other sports, resulting in an overrepresentation of athletes born in the last months before the cut-off date in less popular sports, known as inverse or reverse RAE [20, 21, 29], similar to what may occur in sports in which physicality is not as important (e.g., rhythm gymnastics, aerobics, dance). The present research extends beyond the particularities of high-level popular sports by examining RAE in the lowest grassroots level to ascertain when RAE begins in various competitive sports. Moreover, we aimed to provide a wider view of youth competitive sports with different features, including various sports that may give insight to shuttling of athletes from sport to sport.

To achieve this, we investigated presence of the RAE in male and female athletes aged 9–14 years engaged in competitive sports within Bizkaia, Spain (1,151,905 inhabitants). In Bizkaia, structured and official competitions start at 9 years of age. We included various types of sports, such as team and individual sports; more popular and less popular sports; internationally known, local, and traditional sports; and sports arranged into weight categories. We also included chess, which is considered a “mind sport” with no physicality advantage. In Bizkaia, sports clubs organize and conduct training sessions, and organization of the competitions is supervised by a provincial council. Most youth sports have only one league, but the most popular sports have more than one league depending on the level of play. This allowed us to investigate the RAE from two perspectives: the viewpoint of sports in which children participate without any clear selection (one league), and the viewpoint of sports with a potential selection process (sports with two or more leagues). We included both males and females to individually study each group and to compare sexes. We hypothesized that the RAE would be evident in males participating in the most popular sports and less evident in females and in culturally less popular disciplines.

## Methodology

### Study population and sports league characteristics

We analyzed the dates of birth of 38,381 youth sports participants aged 9–14 years: 13,467 girls (34.9%) and 24,914 boys (65.1%). This included a total of 37 sports: 36 sports for boys and 34 for girls. Participants trained during the week and had competitions on weekends. In team sports, the competitions ran in a league format. Individual sports had competitions along the season, with a final championship at the end of the season. All sports had regional levels. Most sports were organized in two-year age bands; however, in sports with many participants, some teams were based on one-year age bands.

Football was divided in outdoor and indoor football for both sexes. Multisport was a discipline in which athletes participated in a wide variety of sports in the same season one after another, including handball, basketball, football, athletics and swimming.

Most sports had only one league, although a few sports with a large number of participants had more than one league. For boys, outdoor football had three leagues: 1^st^ league, 2^nd^ league, and 3^rd^ league (named “*rendimiento*”, “*competición*”, and “*participación*”, respectively). The 1^st^ league was for 13–14-year-olds only.

In basketball, in both sexes there was a unique league for 9–11-year-olds, and older ages had two leagues: 1^st^ league and 2^nd^ league (“*rendimiento*” and “*competición*”, respectively). These leagues were defined by competitiveness of the teams, with the 1^st^ league being the most competitive and the 2^nd^ and 3^rd^ leagues the least competitive. Basque pelota had four leagues for boys: A (least competitive), B, C, and D (most competitive). The complete list of sports and number of participants by age group are shown in S1 Table for boys and S2 Table for girls.

Due to the low numbers of boys participating in some sports [i.e., rhythmic gymnastics (n = 27), artistic gymnastics (n = 15), trampolining (n = 15), artistic skating (n = 13), aerobics (n = 1), and synchronized swimming (n = 1)], these sports were considered together as a single group titled “gymnastics” (n = 72).

The Ethics Committee of the University of the Basque Country (UPV/EHU) for Research on Human Subjects approved this study. All data were anonymized for the research study.

All sports participants in Bizkaia county must have a license, for which they must supply basic information including date of birth. Thus, we used these data to compare the dates of birth of sports participants to the distribution of general births occurring in Bizkaia in the same year using information from the Eustat Institute for Statistics [32]. Participant dates of birth were first analyzed according to the month of birth. The cut-off date for all sports was January 1. Thus, the year was divided into four quarters for all sports (Q1: January 1–March 31, Q2: April 1–June 30, Q3: July 1–September 30, and Q4: October 1– December 31).

### Statistical analyses

A χ^2^ goodness-of-fit test was used to assess differences between the distributions of observed and expected birth dates. The distribution of expected birth dates was based on the distribution of live births in Bizkaia in the same year [32], following previous studies [13]. The effect size Cramer’s V was measured, considering 0.06, 0.17, and 0.29 as small, medium, and large effect sizes (ES), respectively [33]. Standardized residuals (SR) were determined, with a value of ≥1.96 indicating an overrepresentation and a value of ≤-1.96 indicating an underrepresentation of participants compared to the general population (*p* < 0.05) [28].

Odds ratios (OR) and 95% confidence intervals were calculated to determine the odds of a boy/girl born in January playing in the highest leagues of football (boys) or basketball (girls) compared to the odds of a boy/girl born in each remaining month.

To analyze age as a continuous variable [34], the number of days between the day of birth and December 31 was calculated. This new variable was named “relative age”, and the medians and interquartile ranges (IQR; 25^th^ percentile–75^th^ percentile) were calculated. Relative age was compared among the different leagues as well as different age groups using Wilcoxon and Mann–Whitney U tests.

To give a wide picture of the RAE in all youth competitive sports, we compared the dates of births of all athletes with the actual births in the complete sample (Table 1 for boys, Table 4 for girls). Then analysis was performed within each age group (S3–S8 Tables for boys, S9–S14 Tables for girls), of which statistically significant differences are shown in Table 2 for boys and Table 5 for girls. Additionally, comparison of the median age between leagues and age groups of boys participating in football is shown in Table 3.

**Table 1:** Descriptive statistics of the birth distribution of all male sport participants (9–14 years old) and the general population.

		Birth quarters							Standardized residuals				Relative age (days)	
	n	Q1 n (%)	Q2 n (%)	Q3 n (%)	Q4 n (%)	χ2	p	V	Q1	Q2	Q3	Q4	Median	IQR
Football	14438	3734(25.9)	3831(26.5)	3473(24.1)	3400(23.5)	44.411	***	0.06	4.22	2.25	-2.51	-3.90	193.0	97.0–278.0
Indoor	2038	499(24.5)	527(25.9)	511(25.1)	501(24.6)	0.449	ns	0.01	0.32	0.26	-0.04	-0.53	179.0	97.0–278.0
3^rd^ league	8289	1999(24.1)	2125(25.6)	2056(24.8)	2109(25.4)	0.531	ns	0.01	-0.04	0.09	-0.53	0.48	174.0	98.0–278.5
2^nd^ league	3048	853(27.9)	876(28.7)	700(23)	619(20.3)	64.808	***	0.15	4.31	3.44	-2.35	-5.34	201.0^Ψ^^T^	94.5–273.0
1^st^ league	1059	383(36.2)	301(28.4)	204(19.3)	171(16.1)	115.507	***	0.33	7.94	1.82	-3.80	-5.88	227.0^Ψ^^T^^S^	138.0–295.0
Basketball	1845	478(25.9)	488(26.4)	448(24.3)	431(23.4)	5.852	ns	0.06	1.56	0.74	-0.70	-1.53	191.0	98.0–278.5
2^nd^ league	1657	421(25.4)	440(26.5)	406(24.5)	390(23.5)	3.727	ns	0.05	1.05	0.78	-0.49	-1.32	190.0	97.0–276.0
1^st^ league	188	57(30.3)	48(25.5)	42(22.3)	41(21.8)	4.396	ns	0.15	1.79	-0.14	-0.73	-0.88	204.0	102.0–300.5
Athletics	1149	283(24.6)	303(26.4)	289(25.2)	274(23.8)	1.203	ns	0.03	0.36	0.52	0.06	-0.88	186.0	94.5–273.0
Basque pelota	964	259(26.9)	223(23.1)	269(27.9)	213(22.1)	11.905	**	0.11	1.70	-1.53	1.74	-1.92	183.5	101.0–282.7
D	116	29(25)	24(20.7)	36(31)	27(23.3)	2.917	ns	0.16	0.19	-1.10	-1.07	-0.37	162.0	97.0–288.0
C	143	32(22.4)	41(28.7)	38(26.6)	32(22.4)	1.285	ns	0.09	-0.34	0.66	0.33	-0.67	185.0	86.0–268.5
B	542	152(28)	124(22.9)	142(26.2)	124(22.9)	6.382	ns	0.11	1.83	-1.27	0.51	-1.11	187.0	101.0–282.0
A	64	19(29.7)	13(20.3)	21(32.8)	11(17.2)	4.652	ns	0.27	1.03	-0.97	1.25	-1.25	186.0	138.0–295.0
Trad sport	805	202(25.1)	203(25.2)	183(22.7)	217(27)	0.368	ns	0.02	0.57	-0.21	-1.40	0.98	184.0	85.0–276.0
Taekwondo	717	157(21.9)	191(26.6)	166(23.2)	203(28.3)	5.682	ns	0.09	-1.22	0.59	-1.04	1.64	179.0	76.5–266.0
Chess	678	172(25.4)	160(23.6)	185(27.3)	161(23.7)	3.316	ns	0.07	0.62	-0.99	1.15	-0.76	180.0	95.0–276.2
Swimming	650	166(25.5)	166(25.5)	178(27.4)	140(21.5)	5.302	ns	0.09	0.72	-0.08	1.17	-1.87	190.0	105.0–275.5
Handball	638	157(24.6)	171(26.8)	152(23.8)	158(24.8)	0.885	*	0.04	0.24	0.63	-0.63	-0.24	188.0	92.0–272.0
Karate	424	122(28.8)	97(22.9)	108(25.5)	97(22.9)	5.908	ns	0.12	1.98	-1.06	0.19	-0.97	192.5	103.0–288.7
Hockey	346	88(25.4)	83(24)	83(24)	92(26.6)	1.030	ns	0.05	0.44	-0.53	-0.43	0.54	181.0	84.0–276.0
Cycling	328	88(26.8)	67(20.4)	88(26.8)	85(25.9)	4.859	ns	0.12	1.01	-1.85	0.66	0.22	174.0	88.2–281.7
Judo	295	76(25.8)	76(25.8)	81(27.5)	62(21)	3.009	ns	0.10	0.59	0.12	0.81	-1.39	188.0	106.0–280.0
Rugby	265	56(21.1)	76(28.7)	76(28.7)	57(21.5)	4.751	ns	0.13	-1.00	0.97	1.10	-1.22	182.0	105.0–263.0
Multisport	235	65(27.7)	62(26.4)	52(22.1)	56(23.8)	2.264	ns	0.10	1.06	0.26	-0.91	-0.39	193.0	94.0–281.0
Water polo	174	41(23.6)	53(30.5)	41(23.6)	39(22.4)	2.331	ns	0.12	-0.15	1.19	-0.45	-0.75	200.5	102.0–271.5
Tennis	151	49(32.5)	33(21.9)	32(21.2)	37(24.5)	6.092	ns	0.20	2.17	-0.96	-0.97	-0.16	195.0	93.0–293.0
Rowing	149	39(26.2)	41(27.5)	38(25.5)	31(20.8)	1.615	ns	0.10	0.50	0.49	0.16	-0.99	196.0	103.5–281.5
Triathlon	129	25(19.4)	18(26.4)	18(28.7)	21(25.6)	1.907	ns	0.12	-1.08	-1.22	-1.04	-0.42	172.0	89.5–264.5
Padel	87	21(24.1)	20(23)	23(26.4)	23(26.4)	0.346	ns	0.06	0.00	-0.43	0.21	0.21	169.0	86.0–273.0
Baseball	86	21(24.4)	19(22.1)	23(26.7)	23(26.7)	0.589	ns	0.08	0.22	-0.64	0.21	0.21	170.0	68.7–268.2
Table tennis	73	25(34.2)	13(17.8)	20(27.4)	15(20.5)	5.592	ns	0.28	1.65	-1.38	0.47	-0.71	185.0	110.5–296.5
Gymnastics^¥^	72	15(20.8)	16(22.2)	18(25)	23(31.9)	1.953	ns	0.16	-0.49	-0.47	-0.23	1.18	158.5	65.5–256.0
Volleyball	60	24(40)	10(16.7)	13(21.7)	13(21.7)	8.697	*	0.38	2.32	-1.29	-0.52	-0.52	223.5	99.7–312.0
Frontenis	35	7(20)	10(28.6)	6(17.1)	12(34.3)	2.406	ns	0.26	-0.35	0.33	-1.00	1.00	229.0	132.0–316.0
Skating-races	31	10(32.3)	6(19.4)	5(16.1)	10(32.3)	2.929	ns	0.31	0.71	-0.71	-1.06	0.71	142.0	80.0–252.0
Climbing	23	4(17.4)	8(34.8)	5(21.7)	6(26.1)	1.305	ns	0.24	-0.82	0.82	-0.41	0.45	145.0	68.0–218.0
Boxing	18	7(38.9)	4(22.2)	2(11.1)	5(27.8)	3.154	ns	0.42	1.50	-0.45	-1.00	0.50	228.0	172.0–289.2
Skateboard	17	4(23.5)	4(23.5)	7(41.2)	2(11.8)	2.996	ns	0.42	-0.45	-0.45	1.50	-1.00	229.0	132.0–316.0
Canoeing	17	5(29.4)	3(17.6)	5(29.4)	4(23.5)	0.759	ns	0.21	0.50	-0.50	0.50	-0.45	240.0	117.0–327.0
Archery	15	3(20)	6(40)	4(26.7)	2(13.3)	2.175	ns	0.38	-0.50	1.00	0.58	-1.00	218.0	90.0–324.0
Total	24914	6403(25.7)	6477(26)	6110(24.5)	5924(23.8)	49.722	***	0.04	5.03	1.30	-1.83	-4.41	190.0	96.0–277.0
Births Gen Pop	29462	7111(24.1)	7538(25.6)	7395(25.1)	7418(25.2)									

n: number of players; Q: birth quarter; V: Cramer’s V; IQR: interquartile range (25^th^ and 75^th^ percentiles are shown); SR: standardized residuals (comparison of the distribution between participants and the general population); Trad sport: traditional sport; Gymnastics^¥^: includes rhythmic gymnastics (n = 27), sports gymnastics (n = 15), trampolining (n = 15), figure skating (n = 13), aerobics (n = 1), synchronized swimming (n = 1); Gen Pop: general population; ns: not significant; χ2: chi squared

*p<0.05

**p<0.01

***p<0.001

^Ψ^p<0.001 statistically significant differences vs. indoor

^T^p<0.001 statistically significant differences vs. 3^rd^ league

^S^p<0.001 statistically significant differences vs. 2^nd^ league

**Table 2:** Descriptive statistics of the birth distribution of male sport participants divided by age group (only statistically significant results are shown).

		Total (n)	Q1%	Q2%	Q3%	Q4%	χ2	P	V	Standardized residuals			
										Q1	Q2	Q3	Q4
9 y	Total	4061	25.6	27.3	24.4	22.7	17.887	<0.001	0.07	1.56	2.02	-0.16	-0.36
	All football	2233	25.8	27.9	24.0	22.4	13.531	0.004	0.08	1.33	1.96	-0.51	-2.77
	2^nd^ L football	261	34.9	32.2	21.8	11.1	37.636	<0.001	0.38	3.38	2.08	-0.88	-4.55
10 y	Total	4690	25.6	26.5	24.7	23.3	18.429	<0.001	0.06	2.74	1.48	-1.72	-2.40
	All football	2608	25.5	27.4	24.3	22.8	15.722	0.001	0.08	2.06	1.94	-1.65	-2.23
	2^nd^ L football	494	30.6	30.0	21.9	17.6	29.023	<0.001	0.24	3.14	1.96	-1.77	-3.25
11 y	2^nd^ L football	553	27.5	31.1	21.9	19.5	19.025	0.000	0.19	1.74	2.52	-1.68	-2.55
	Athletics	221	23.1	22.6	33.5	20.8	7.922	0.048	0.19	-0.27	-0.93	2.41	-1.21
12 y	All football	2506	25.7	26.4	23.4	24.5	10.488	0.015	0.06	2.09	1.07	-1.79	-1.30
	1^st^ L football	280	42.5	26.1	15.4	16.1	63.084	0.000	0.47	6.52	0.24	-3.32	-3.28
	Trad sport	131	29.0	19.8	16.0	35.1	11.915	0.008	0.30	1.26	-1.22	-2.09	2.06
13 y	1^st^ L football	367	32.7	28.9	20.7	17.7	25.500	<0.001	0.26	3.54	1.27	-1.76	-2.90
	Chess	44	15.9	13.6	43.2	27.3	9.244	0.024	0.46	-0.95	-1.51	2.41	0.30
14 y	All football	2112	27.2	26.2	23.6	23.0	14.005	<0.001	0.08	1.33	0.43	-0.40	-1.41
	1^st^ L football	412	35.0	29.6	20.6	14.8	33.676	<0.001	0.29	3.69	1.55	-1.41	-3.98
	Taekwondo	79	25.3	11.4	24.1	39.2	11.793	0.008	0.39	-0.22	-2.46	0.00	2.75
	Gymnastics	12	16.7	8.3	8.3	66.7	11.077	0.011	0.96	-0.58	-1.15	-1.15	2.89

n: number of players; Q: birth quarter; V: Cramer’s V; y: years; L: league; Trad sport: traditional sport

**Table 3:** Relative age of male football participants according to age group and league [medians and interquartile ranges (25th– 75th percentiles)].

	Football (all)	Indoor	3^rd^ league	2^nd^ league	1^st^ league
9 y	195.0 (103.0–277.0)	172.0 (89.0–268.2)	195.0 (275.0–98.0)	240.0 (315.0–151.5)^III^^TTT^	
10 y	194.0 (101.0–276.0)	191.0 (95.0–277.0)	186.0 (271.0–95.0)	215.0 (290.0–123.7)^II^^TTT^	
11 y	189.0 (94.0–277.0)	186.0 (95.0–267.0)	181.0 (276.0–86.5)	210.0 (283.0–116.5)^##^^I^^TT^	
12 y	193.0 (93.0–277.0)	187.0 (83.5–272.0)	180.0 (267.0–88.0)^#^	193.5 (276.5–102.2)^###^^&^^T^	254.5 (314.0–148.5)^III^^TTT^^SSS^
13 y	188.0 (97.0–277.0)	160.5 (94.0–280.0)	174.0 (270.0–88.0)^#^	199.0 (276.2–99.7)^###^^&^^T^	221.0 (293.0–132.0)^Ψ^^II^^TTT^^SS^
14 y	196.5 (99.0–284.0)	190.0 (103.0–297.0)	173.0 (266.0–81.5)^##^^&^	204.0 (286.0–101.2)^###^^TTT^	230.0 (299.0–138.0)^I^^TTT^^SSS^
**All**	193.0 (97.0–278.0)	179.0 (97.0–278.0)	174.0 (278.5–98.0)	201.0 (273.0–94.5)^III^^TTT^	227.0 (295.0–138.0) ^III^^TTT^^SSS^

y: years old

Comparisons between years (within the same level of competition)

^#^p<0.05

^##^p<0.01

^###^p<0.001 statistically significant differences vs. 9 years old

^&^p<0.05, ^&&^p<0.01, ^&&&^p<0.001 statistically significant differences vs. 10 years old

^Ψ^p<0.05, ^ΨΨ^p<0.01, ^ΨΨΨ^p<0.001 statistically significant differences vs. 12 years old

Comparisons between football leagues (within the same year)

^I^p<0.05

^II^p<0.01

^III^p<0.001 statistically significant differences vs. indoor

^T^p<0.05

^TT^p<0.01

^TTT^p<0.001 statistically significant differences vs. 3^rd^ league

^S^p<0.05

^SS^p<0.01

^SSS^p<0.001 statistically significant differences vs. 2^nd^ league

**Table 4:** Descriptive statistics of the birth distribution of female sport participants and the general population.

		Birth quarters							Standardized residuals				Relative age (days)	
	n	Q1 n (%)	Q2 n (%)	Q3 n (%)	Q4 n (%)	χ2	p	V	Q1	Q2	Q3	Q4	Median	IQR
Basketball	3780	1020(27)	1028(27.2)	880(23.3)	852(22.5)	32.474	***	0.09	3.47	1.99	-1.74	-3.67	198.0	102.3–282.0
2^nd^ league	3534	937(26.5)	961(27.2)	831(23.5)	805(22.8)	24.081	***	0.08	2.77	1.93	-1.42	-3.26	196.0	102.0–280.0
1^st^ league	246	83(33.7)	67(27.2)	49(19.9)	47(19.1)	15.772	***	0.25	2.97	0.50	-1.54	-2.02	222.5^SS^	119.7–305.0
Rhythmic gymn	1434	350(24.4)	385(26.8)	333(23.2)	366(25.5)	2.222	ns	0.04	0.16	0.94	-1.12	-0.05	189.0	90.0–273.0
Handball	1307	334(25.6)	332(25.4)	348(26.6)	293(22.4)	7.983	*	0.08	1.01	-0.11	1.39	-2.24	188.0	101.0–277.0
Football	1262	344(27.3)	338(26.8)	281(22.3)	299(23.7)	10.307	*	0.09	2.17	0.83	-1.76	-1.28	201.0	96.0–285.0
Indoor	178	55(30.9)	36(20.2)	47(26.4)	40(22.5)	6.139	ns	0.19	1.83	-1.34	0.45	-0.75	202.5	92.2–283.7
Outdoor	1084	289(26.7)	302(27.9)	234(21.6)	259(23.9)	10.349	*	0.10	1.67	1.50	-2.08	-1.08	189.0	97.7–293.2
Athletics	1179	307(26.0)	317(26.9)	272(23.1)	283(24)	4.813	ns	0.06	1.30	0.92	-1.17	-1.04	197.0	96.0–279.0
Volleyball	734	181(24.7)	201(27.4)	182(24.8)	170(23.2)	2.667	ns	0.06	0.22	0.95	0.07	-1.24	196.0	97.8–273.3
Swimming	636	166(26.1)	160(25.2)	152(23.9)	158(24.8)	1.265	ns	0.04	0.97	-0.16	-0.40	-0.31	186.0	92.0–280.0
Trad sport	590	140(23.7)	165(28.0)	138(23.4)	147(24.9)	1.900	ns	0.06	-0.25	1.14	-0.66	-0.33	191.0	91.8–272.0
Taekwondo	438	109(24.9)	99(22.6)	105(24.0)	125(28.5)	3.199	ns	0.09	0.29	-1.23	-0.29	1.23	178.0	79.8–273.5
Chess	249	59(23.7)	57(22.9)	61(24.5)	72(28.9)	1.826	ns	0.09	-0.13	-0.88	-0.13	1.00	174.0	76.0–270.5
Hockey	207	39(18.8)	63(30.4)	50(24.2)	55(26.6)	4.504	ns	0.15	-1.56	1.37	-0.14	0.27	182.0	88.0–249.0
Karate	190	43(22.6)	45(23.7)	57(30.0)	45(23.7)	2.881	ns	0.12	-0.44	-0.43	1.46	-0.43	169.0	96.3–267.5
Artistic skating	180	34(18.9)	52(28.9)	46(25.6)	48(26.7)	3.031	ns	0.13	-1.51	0.88	0.30	0.29	179.5	85.3–256.8
Basque pelota	137	44(32.1)	38(27.7)	23(16.8)	32(23.4)	7.519	ns	0.23	1.91	0.51	-1.89	-0.51	219.0	98.5–299.5
Skate-racing	115	21(18.3)	33(28.7)	35(30.4)	26(22.6)	4.050	ns	0.19	-1.32	0.74	1.32	-0.56	171.0	100.0–242.0
Aerobic	111	27(24.3)	23(20.7)	33(29.7)	28(25.2)	2.16	ns	0.14	0.20	-0.94	-0.81	-0.19	175.0	91.0–270.0
Multisport	97	23(23.7)	20(20.6)	24(24.7)	30(30.9)	2.033	ns	0.14	-0.20	-1.00	0.21	1.00	155.0	78.0–272.0
Judo	95	26(27.4)	17(17.9)	24(25.3)	28(29.5)	3.157	ns	0.18	0.63	-1.43	0.21	0.82	177.0	76.0–287.0
Padel	92	18(19.6)	24(26.1)	25(27.2)	25(27.2)	1.154	ns	0.11	-0.85	0.21	0.42	0.20	181.0	80.0–254.3
Tennis	88	20(22.7)	27(30.7)	20(22.7)	21(23.9)	1.221	ns	0.12	-0.22	0.83	-0.43	-0.21	191.0	97.3–263.0
Triathlon	86	24(27.9)	22(25.6)	18(20.9)	22(25.6)	0.979	ns	0.11	0.65	0.22	-0.65	0.22	206.0	85.0–283.5
Baseball	73	13(17.8)	20(27.4)	19(26.0)	21(28.8)	1.681	ns	0.15	-1.18	0.23	0.24	0.46	159.0	81.5–241.0
Synchronized sw	72	21(29.2)	20(27.8)	20(27.8)	11(15.3)	4.121	ns	0.24	0.97	0.47	0.47	-1.65	209.0	115.3–283.0
Cycling	69	13(18.8)	19(27.5)	14(20.3)	23(33.3)	3.106	ns	0.21	-0.97	0.24	-0.73	1.18	166.0	66.0–267.0
Water polo	50	14(28.0)	8(16.0)	17(34)	11(22)	4.083	ns	0.29	0.58	-1.39	1.44	-0.55	178.0	94.0–297.0
Rugby	45	14(31.1)	8(17.8)	11(24.4)	12(26.7)	1.973	ns	0.21	0.90	-0.90	-0.29	0.30	172.0	55.0–290.5
Rowing	29	8(27.6)	7(24.1)	9(31)	5(17.2)	1.415	ns	0.22	0.38	-0.35	0.76	-0.76	190.0	114.0–281.5
Canoeing	27	9(33.3)	7(25.9)	5(18.5)	6(22.2)	1.464	ns	0.23	0.76	0.41	-0.76	-0.38	237.0	111.0–292.0
Artistic gymn	26	8(30.8)	7(26.9)	5(19.2)	6(23.1)	0.858	ns	0.18	0.82	0.41	-0.41	-0.38	189.0	93.5–292.0
Climbing	21	3(14.3)	9(42.9)	6(28.6)	3(14.3)	4.486	ns	0.46	-0.89	1.79	0.45	-0.89	193.0	162.5–264.0
Archery	16	4(25.0)	2(12.5)	7(43.8)	3(18.8)	3.714	ns	0.48	0.58	-1.00	1.50	-0.50	162.5	102.5–277.3
Skiing	13	1(7.7)	5(38.5)	4(30.8)	3(23.1)	2.538	ns	0.44	-1.15	1.15	0.58	-0.50	158.0	100.0–229.0
Trampolining	10	4(40.0)	1(10.0)	2(20.0)	3(30.0)	2.143	ns	0.46	1.41	-1.15	-0.58	0.71	196.5	51.3–292.0
Table tennis	9	3(33.3)	2(22.2)	2(22.2)	2(22.2)	0.410	ns	0.21	0.71	-0.58	-0.58	-0.58	187.0	50.0–329.5
Total Participants	13467	3444(25.6)	3560(26.4)	3226(24.0)	3237(24.0)	29.771	***	0.05	3.24	2.03	-1.67	-3.53	191.0	95.0–277.0
Births Gen Pop	27685	6702(24.2)	7074(25.5)	6836(25.5)	7073(25.5)	32.474								

n: number of players; Q: birth quarter; IQR: interquartile range (25^th^ and 75^th^ percentiles are shown); V: Cramer’s V; SR: standardized residuals; sw: swimming; Gymn: gymnastics; Trad sport: traditional sport; Gen Pop: general population; ns: not significant.; χ2: chi squared

*p<0.05

**p<0.01

***p<0.001

^SS^p<0.001 statistically significant differences vs. 2^nd^ league

**Table 5:** Descriptive statistics of the birth distribution of female sport participants divided by age group (only statistically significant results are shown).

		Total (n)	Q1%	Q2%	Q3%	Q4%	χ2	p	V	Standardized residuals			
										Q1	Q2	Q3	Q4
10 y	Total	2503	25.4	27.5	22.7	24.3	8.880	0.031	0.06	1.47	1.49	-1.66	-1.49
	Basketball	725	28.1	27.7	22.5	21.7	11.711	0.008	0.13	2.27	0.87	-1.05	-2.13
11 y	Total	2627	25.8	26.5	24.2	23.6	15.435	0.001	0.08	1.50	2.34	-1.31	-2.45
	Handball	245	31.4	17.6	22.0	29.0	11.597	0.009	0.22	2.19	-2.08	-1.02	0.88
	Karate	37	10.8	13.5	45.9	29.7	10.835	0.013	0.54	-1.67	-1.33	2.67	0.32
12 y	Total	2627	25.2	27.4	24.7	22.7	11.191	0.022	0.07	1.44	1.53	-0.39	-2.56
	All basketball	780	27.3	26.9	24.6	21.2	9.600	0.022	0.11	1.98	0.56	-0.29	-2.28
	1^st^ league basketball	67	32.8	32.8	22.4	11.9	8.448	0.038	0.36	1.50	1.21	-0.49	-2.18
13 y	Handball	258	25.6	21.3	33.3	19.8	11.435	0.010	0.21	0.38	-1.24	2.60	-1.74

n: number of players; Q: birth quarter; V: Cramer’s V; y: years

Statistical analysis was performed using IBM SPSS statistics software (v 22.0). The level of significance was set at *p* < 0.05.

## Results

The most popular sport for boys was football (57.4%), followed by basketball (7.3%) and athletics (4.5%). For girls, the most popular sports were basketball (28.1%), rhythmic gymnastics (10.6%), and handball (9.7%).

The distribution of birth dates of all male participants was significantly different from that of the general population (*p*<0.001), with a significant overrepresentation in Q1 and underrepresentation in Q4 (Table 1). Among all football players (small ES) as well as among those in the 2^nd^ (small ES) and 1^st^ (large ES) leagues, there was an overrepresentation of birth dates in Q1 and Q2 and an underrepresentation in Q3 and Q4. Moreover, players in the 1^st^ and 2^nd^ leagues were relatively older (*p*<0.001) than players in indoor and 3^rd^ league football. Players in the performance football league were relatively older than players in the competition league (*p*<0.001).

The distribution of birth dates among participants in Basque pelota (*p*<0.01), handball (*p*<0.05), and volleyball (*p*<0.05) was also significantly different from that of the general population.

Male participant birth dates were analyzed by year group (S3–S8 Tables). In the 9- and 10-year-old groups, the distribution of birth dates of all participants (*p*<0.001, small ES) as well as all football (*p*<0.01) and football 2^nd^ league participants (*p*<0.001) was significantly different from that of the general population (Table 2). There was an overall overrepresentation of birth dates in Q1 and underrepresentation in Q4 in these leagues.

In the 11-year-old group, significant differences in birth date distribution (moderate ES) were observed for 2^nd^ league football (*p*<0.001) and athletics (*p*<0.05).

In the 12-year-old group, there was a significant difference in the birth date distribution for all football (*p*<0.05), 1^st^ football league (*p*<0.001), and traditional sport (*p*<0.01) participants. In the 13-year-old group, a large effect size difference in the distribution of birth dates was found for 1^st^ football league (*p*<0.001) and chess (*p*<0.05) compared to that of the general population.

In the 14-year-old group, there was a significant difference in the birth date distribution of all football (*p*<0.001), 1^st^ football league (*p*<0.001), taekwondo (*p*<0.01), and gymnastics (*p*<0.05).

Among males, 9-year-old players had a higher relative age than 11-year-old players in the 2^nd^ football league (*p*<0.01) as well as 12-, 13-, and 14-year-old players in both the 3^rd^ (*p*<0.05 to *p*<0.01) and 2^nd^ leagues (*p*<0.001) (Table 3). The 10-year-old players had a higher relative age (*p*<0.05) than 12- (2^nd^ league), 13- (2^nd^ league), and 14- (3^rd^ league) year-old players. In the 1^st^ league, the 12-year-old group had a higher relative age than the 13-year-old group (*p*<0.05).

The median relative age of participants in indoor football was lower (*p*<0.05 to *p*<0.001) than that of players in the 2^nd^ (total group and 9-, 10-, and 11-year-olds) and 1^st^ leagues (total group and 12-, 13-, and 14-year-olds). Players in the 3^rd^ league were relatively younger than players in the 2^nd^ and 1^st^ leagues in all age groups (*p*<0.05 to *p*<0.001). Players in the 2^nd^ league were relatively younger (*p*<0.01 to *p*<0.001) than players in the 1^st^ leagues (total group and 12-, 13-, and 14-year-olds).

For girls (Table 4), a significantly different distribution of birth dates compared to the general population occurred in the total sample (*p*<0.001), in all basketball (*p*<0.001), and in 2^nd^ (*p*<0.001) and 1^st^ (*p*<0.001) basketball leagues, with an overrepresentation of players born in Q1 and an underrepresentation of players born in Q4. Moreover, in basketball, players in the 1^st^ league had higher relative age than players in the 2^nd^ league (*p*<0.05).

The distribution of birth dates also significantly differed in handball (*p*<0.01), all football (*p*<0.05), and outdoor football (*p*<0.05).

Female participant birth dates were also analyzed by year group (S9–S14 Tables). The birth quarter distribution in the 10-year-old group was significantly different from that of the general population in the total group (*p*<0.05) and for basketball (*p*<0.01) (Table 5). In the 11-year-old group, there was a significant difference in the birth date distribution for the total group (*p*<0.001), handball (*p*<0.01), and karate (*p*<0.05). Significant differences in the distribution of births were observed in 12-year-olds in the total group (*p*<0.05), all basketball (*p*<0.05), and 1st basketball league (11.9% players born in Q4, large ES, *p*<0.05). Finally, in the 13-year-old group, the birth date distribution differed for handball (*p*<0.05), where players born in Q3 were overrepresented. In summary, except for karate in the 11-year-old group, with 45.9% players born in Q3 (large ES), the trend was toward a large percentage of participants born in Q1 and small percentage in Q4.

Regarding the median relative age of female 1^st^ and 2^nd^ league participants in basketball, unlike male football, the only statistically significant difference was that players of the 1^st^ league were relatively older than players of the 2^nd^ league in the 13-year-old age group (*p*<0.05). Otherwise, there were not statistically significant differences among leagues nor among age groups (S9–S14 Tables).

Regarding the comparison of relative age between sexes, females had higher relative age than males for 10-year-old basketball players (*p*<0.05) and 12-year-old taekwondo participants (*p*<0.01), while males had higher relative age for 13-year-old tennis players (*p*<0.05) and 14-year-old volleyball players (*p*<0.05) (S3–S14 Tables).

**Fig 1:**
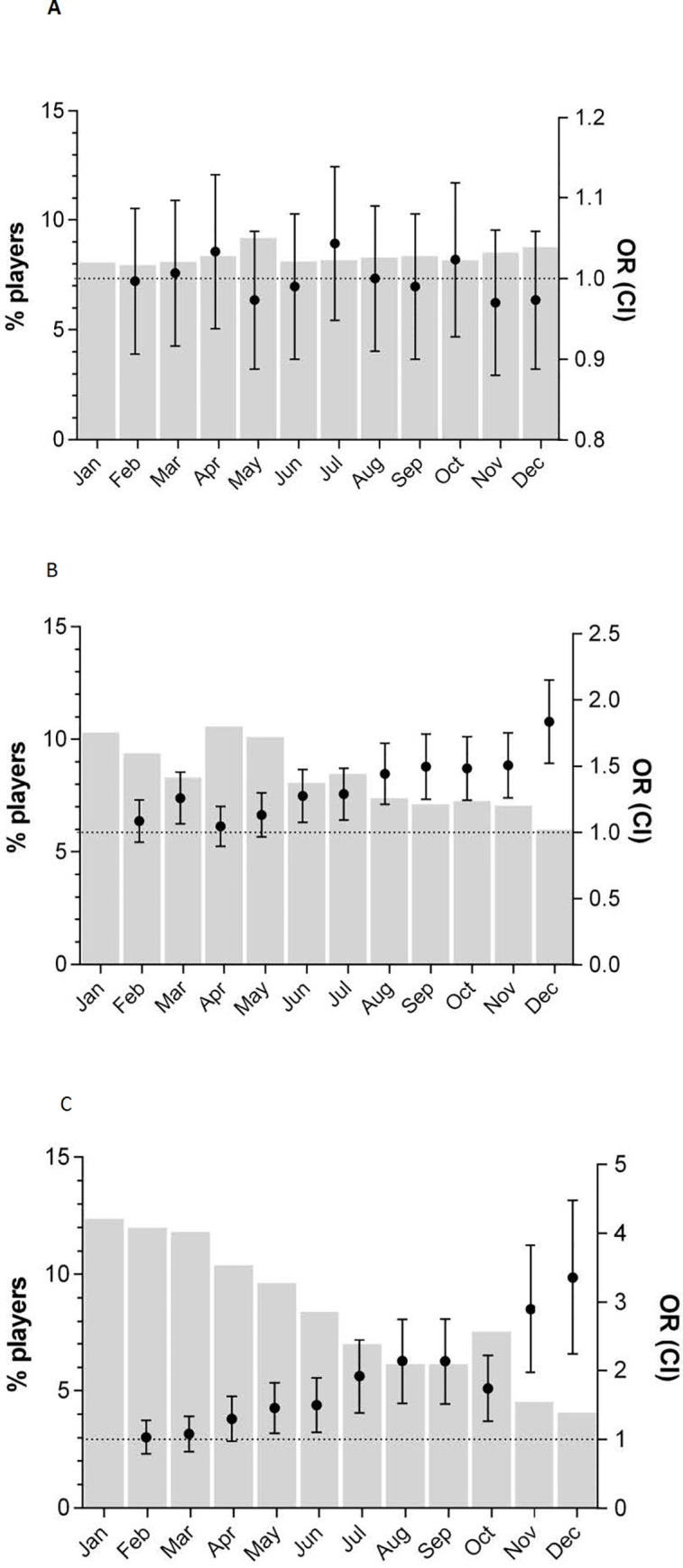
Percentage of male football players born in each month. The percentage of male football players born in each month (gray bars) is shown on the left axis; the right axis displays the odds ratio (OR) (95% confidence interval) of a boy born in January compared to that of a boy born in the remaining months to participate in football at 3^rd^ league (A), 2^nd^ league (B), and 1^st^ league (C) levels.

The odds of a boy born in January playing in the 2^nd^ football league were not statistically different from the odds of a boy born in February, March, or April; however, from June onward, each monthly odds comparison was statistically significant (i.e., June OR = 1.27; 95% CI = 1.08–1.48). The level of significance increased steadily up to December (OR = 1.82; 95% CI = 1.53–2.16). The differences in the 1^st^ league were larger, with the odds of a boy born in January playing being statistically greater than those of a boy born in May (OR = 1.42; 95% CI = 1.10–1.83). By December, the odds were even higher (OR = 3.23; 95% CI = 2.30–4.54), meaning that boys born in January were 3.23 times more likely to play in the performance league than boys born in December (Fig 1).

**Fig 2:**
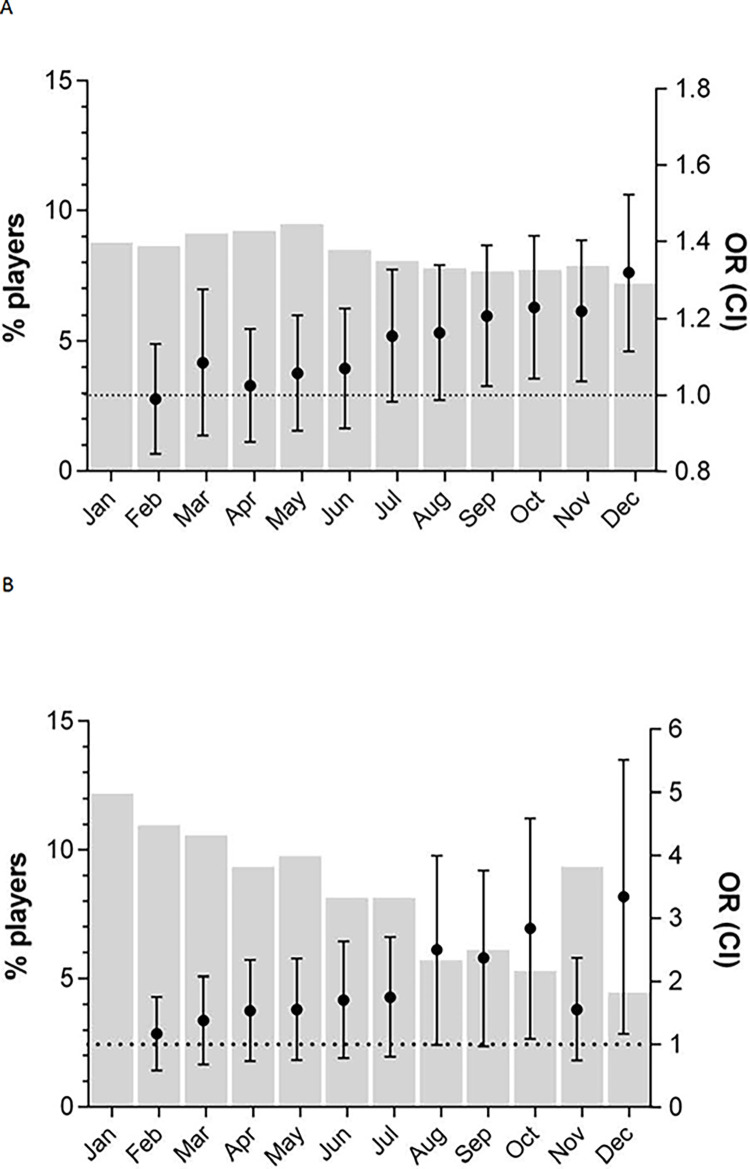
Percentage of female basketball players born in each month. The percentage of female basketball players born in each month (gray bars) is shown on the left axis; the right axis displays the odds ratio (OR) (95% confidence interval) of a girl born in January compared to that of a girl born in each remaining month to participate in basketball at the 2^nd^ league (A) and 1^st^ league (B) levels.

The odds of a girl born in January playing basketball in the 2^nd^ league were statistically larger than the odds of a girl born in September (OR = 1.23; 95% CI = 1.03–1.46), October (OR = 1.25; 95% CI = 1.05–1.49), November (OR = 1.24; 95% CI = 1.04–1.48), or December (OR = 1.36; 95% CI = 1.13–1.62). The odds of a girl born in January playing in the 1^st^ league were statistically larger than those of a girl born in August (OR = 2.21; 95% CI = 1.16–4.18), September (OR = 2.10; 95% CI = 1.13–3.93), October (OR = 2.50; 95% CI = 1.30–4.80), or December (OR = 2.89; 95% CI = 1.44–5.79) (Fig 2).

## Discussion

The overrepresentation of athletes born in the months following the cut-off date, known as the RAE, has primarily been investigated in young and adult male athletes in culturally popular sports and participating in high-level competitions and has frequently been limited to one or only a few sports. Therefore, we aimed to provide a broader insight of the RAE by exploring the birth dates of an entire population of young male and female athletes who participated in a variety of competitive sports in the county of Bizkaia in Spain.

Taking all sports participants into account, we observed an overall RAE in both boys and girls, but with a small effect size. Interestingly, several differences were identified among sexes and sports that merit further attention. As occurs in other countries, participation in sports as well as the selected sports differed by sex [30]. Unquestionably, the prevailing sport for males was football (57.4%), whereas the participation distribution for females was more homogeneous with basketball (28%) followed by rhythmic gymnastics, handball, and football (9%–10%). This distinct participation may have accounted for differences in presence of the RAE and its causes between sexes.

### Analysis of the RAE in grassroots male athletes

In the sport-by-sport analysis, the RAE was almost exclusively present for males in football. In addition, in all males in Basque pelota and handball, there was a small effect RAE with no significant standardized residuals and a large effect in volleyball (Q1) that could not be corroborated by year analysis. In contrast, in two large studies undertaken in the London Youth Games [30] and the Swiss Talent Development Program RAE was confirmed in most analyzed sports [31]. These differences among the studies may be due to the selection processes for sport participation, as the participants of the two last-mentioned studies were either selected to represent their school [30] or were part of a national talent program [31]. The selection hypothesis is the most mentioned cause for the RAE [1–16]. Interestingly, our study could not corroborate the presence of a clear RAE in other sports with more than one league (basketball and Basque pelota), which would *a priori* involve selection of players. Therefore, it is reasonable to think that the underlying reasons for the RAE not only encompass the selection processes of athletes, but are more complex and probably multifactorial, involving a combination of factors in addition to the selection processes.

As mentioned before, physical growth and better functional capacities of relatively older children have been claimed to be the main causes of the RAE [35, 36], particularly in sports in which a large body size and strength are advantageous [1, 2]. Supporting this notion, studies on sports with weight categories [17–20] have not detected the RAE, similar to our results. However, our study did not observe RAE in other sports in which physicality is relevant, such as basketball, handball, or rugby. Therefore, physical attributes and related performance indicators are undeniable factors, but other contributors seem to be just as necessary for RAE to occur.

In this regard, our results indicate that the popularity of a sport (football) is a strong moderator of the RAE, as demonstrated in culturally popular sports in other countries, including rugby in the UK [7], ice hockey in Canada [37], skiing in Austria [12], and handball in Germany [8]. Participation in these sports was lower in our study, 2.5% and 1% of the male participants for handball and rugby, respectively, and therefore it could be assumed that there would not be selection constraints for boys willing to participate in these sports and as a consequence, the birth dates of their players were evenly distributed, and thus no RAE was detected. In addition, particularly in team sports, due to the limited number of spaces on each team, coaches must select a certain number of players and the depth of competition (as represented by the number of participants and number of players selected for a higher competition) may be an additional moderator [2, 8, 30].

Maturity should also be considered when exploring the RAE, specifically around puberty [38, 39]. A closer look at the performance football league demonstrates that the oldest relative age players were those in the 12-year-old group, where 42% were born in Q1, but this percentage decreased in subsequent years. The drop the in relative age may be due to the influence of maturation on the selection of players around puberty, which may be more important than the relative age at other ages [39]. Thus, advanced maturity status may offset some of the disadvantages associated with being younger [40].

### Analysis of the RAE in grassroots female athletes

We found some similarities and discrepancies in the distribution of birth dates of females compared to males. While the RAE almost exclusively occurred in male football participants, particularly at the highest performance levels, in female participants it occurred in three of the four most popular sports (basketball, handball, and football), all of which are team sports. Similarly, RAE has been described in female basketball [6] and football [25] players as young as 7 and 8 years old, respectively. Nonetheless, we did not detect the RAE in female participants in rhythmic gymnastics, the second most popular sport, in agreement with results from Van Rosum [41] in ballet dancers and Baker et al. [42] in figure skaters. Rhythmic gymnastics is an individual sport (no need for coaches and technical staff to identify the best athletes) and an aesthetic sport, where technical staff search for females with small, lean, thin bodies and good flexibility and coordination. Therefore, not merely popularity but its combination with depth of competition and also the physicality of these sports may be responsible for the RAE. Further, other related factors also may be associated.

In the yearly analysis by sport, RAE occurred in two distinct scenarios. On one hand, this phenomenon was linked to basketball, when this sport started to be organized in two leagues (i.e., 12-year-old 1^st^ league). This indicates that popularity of the sport and the selection processes are responsible for the biased distribution of players’ birth dates, similar to what happens with males. On the other hand, a large effect size RAE was observed in female 9-year-old basketball players and also in 11- and 13-year-old handball players. These results are interesting because they cannot be attributed to coach selection or depth of competition. Instead, it is possible that social influences might be involved. One possibility is that parents do not encourage/enroll their relatively younger children in sports with high physical demands, as may be the case for handball, for fear of injury or failure [5]. Additionally, relatively younger players with smaller bodies and lower performances might eschew participation because they perceive that they are not as capable as older players. This effect is referred to as internal or self-selection [1, 2]. Additionally, in the most popular sports, younger players are more likely than older players to drop out, reinforcing presence of the RAE [35, 43, 44].

### Further analysis: The case of chess and the inverse RAE

Chess is of particular interest because it is a mind sport, relying on perception and cognitive skills rather than physical strength [45]. Studies on this discipline are scarce, but the RAE has been described in expert adult chess players [46] and top junior male and female players [47]. Helsen et al. [45] confirmed the presence of the RAE in U8–U20 chess players in the Belgian Championship but failed to demonstrate it in non-elite U12–U20 chess players, supporting the uneven distribution of birth dates observed in the small group of male 13-year-old males in this study—which incidentally indicates an inverse RAE.

Athletes born in the last quarters of the year may take up other less popular sports, resulting in an overrepresentation of athletes born in the last months before the cut-off date, known as inverse RAE [20, 21, 29], observed in the present study in male athletics, traditional sport, chess, taekwondo, and gymnastics across 11-, 12-, 13-, and 14-year-old groups, respectively; which partially corroborates our hypothesis. This inverse RAE has been described in 15–17 years old male French shooters [20] and female table tennis players, fencers and snowboarders of a Swiss talent development program [29]. These sporting activities require high technical skills or aesthetics for performance [29] and physical attributes may not be that important [20], thus relatively younger, smaller and less strong athletes may have an advantage and are more likely to participate and be selected [29]. Moreover, in sports with less attendance, all children who are willing to participate have the opportunity to do so, independent of their relative age, while in more popular sports, there are some obstacles that are difficult for relatively younger players to overcome.

### General implications of the RAE

The overrepresentation of relatively older children involved in the RAE is not the major concern—what is worrisome is the significant and systematic underrepresentation of relatively young participants. If children are not able to participate and enjoy sports from a young age, it will be more difficult for them to take up a sport in adolescence and even harder in adulthood. While birth date was not a strong constraint to participation in most sports in Bizkaia, there were 360 fewer boys and 207 fewer girls born in the last quarter of the year participating in sports relative to the numbers expected from births in the entire population. This underrepresentation was especially concerning in some particular sport contexts, and the implications of these findings are twofold. First, and most importantly, there is a clear discrimination against relatively young children solely on the basis of having been born in certain months of the year. As an illustration, a boy born in January was 1.8-times more likely than a boy born in December to play in the competition football league, and this difference was even larger (3.2-times) in the performance league. The odds of a girl born in January playing basketball were significantly larger than those of a girl born later than July. Therefore, special effort should be made to ensure that all children have the same opportunities to participate in a sport, particularly at young ages (i.e., 9 years old). Second, as talent is born homogeneously across the year, talent may be missed each time a relatively young athlete is not selected, discarded, or drops out of a sport.

### Measures to mitigate the RAE

In the literature, several recommendations have been proposed to avoid the RAE. Our results suggest the RAE occurs because of a combination of different factors and is closely linked to a particular sports context, so all steps should be taken accordingly. In the most popular sports, selection processes should be avoided at the grassroots level and delayed as much as possible, preferentially until post-maturation [1, 48]. If selection procedures are indispensable, they should be improved to prevent the RAE [48]. In this line, changing and rotating the cutoff date has been proposed as a strategy to remove particular selection time points and give all athletes the possibility of being relatively older at some point [1, 2, 4, 26]. Thus, it has been proposed a rotation of the cut-off dates by 3 months between seasons of competition, to ensure players have experience in each quartile position [49]. However, despite being a largely mentioned solution its implementation would be rather complicated in the actual sporting competitions. Also, “birth-day banding”, where athletes move up to their next birthdate group on their birthday, has been successfully applied in the England squash Talent Pathway [50].

Since the origin of the RAE lies on the date of birth of the children and youth, some authors [2, 4] have recommended a different classification system based on the biological age of the participants instead of the chronological age, similar to bio-banding. In order to equalize the advantages of maturity, in bio-banding athletes are grouped according to their maturity status [51]. In younger immature children, classification could be based on body height, weight and size.

Organizing leagues and championships among children of a similar age (i.e., teams made up of children born a maximum of six months apart, or less if the magnitude of the sport allows it) could be possible for very popular sports [50]. Also, different squads may be established for those athletes who are technically at high standard, but who are lacking physical development, in order to give them a better chance for fair competition [2]. Further, a mean compulsory age could be implemented so that coaches equally incorporate relatively younger and older players to teams to provide similar opportunities [1].

Additionally, providing scouts who are responsible for finding talent with information about the relative age of the players being observed helps to avoid the RAE on player selection [1]. Mann et al. (2016) demonstrated that the selection bias was eliminated when scouts were provided with information about the relative age of the players and players wore shirts with numbers corresponding to their relative age while training [52].

In individual sports in which performance is measured by numeric data (e.g., time, distance), individual data can be compared using corrective adjustment procedures, as proposed in athletics [52, 53] and swimming [15, 54]. Interestingly, Abbot et al. [15] found RAEs in 12–14 years old Australian female 100m and 200m Breaststroke swimmers which increased at the highest levels. However, when corrective adjustments procedures were applied the relative age inequalities were removed. Further, these corrective adjustments could also be used for data related to other types of physical condition tests used in team sports, for instance (e.g., yo-yo intermittent test).

Technical staff of the clubs, coaches, and scouts must be aware of relative age and its negative effects [55]. Coaches should avoid instant progress and short time success [56]; instead, they should adopt a long-term vision, giving more emphasis to technical and tactical competences [5, 48] rather than strength, speed, power, and body size, which are tightly related to the growth process.

Further, other factors beyond coaches’ selection seem to be involved in the RAE, so solutions should extend in a broader context as well. Hancock et al. [5] observed a significant overrepresentation of children born in Q1 and underrepresentation in Q4 in children aged 5 years old engaged in ice hockey and suggested that parents might contribute to these early RAEs. As parents may be involved in the enrollment of athletes in sports, they should be provided with appropriate information and education on the sports. To our knowledge no studies have analysed the reasons and the influence of parental decisions to enroll their children in one sport or another on the RAEs, which would be of interest [5].

This study shows that, in addition to all the above measures, it is important to offer an extensive range of sports with various characteristics to promote participation of children and adolescents in physical activity, allowing all children the opportunity to find their preferred sport.

More studies are still necessary to diminish the deleterious effect of relative age. Particularly, programs implementing the proposed measures are crucial. Moreover, broad longitudinal studies should be conducted to discover shifts of athletes between sports in relation to their relative age. In addition, the physical, physiological, social, and psychological mechanisms involved in the continuation and drop-out of relatively young participants should be examined. In this vein, it is interesting the concept of the Relative Age Effect Reversal, which describes the higher sporting level achieved by the relatively younger athletes. The players born at the end of the year and that tended to be neglected in the younger categories, being less often selected for teams, arrive at the adult category with advantageous attributes, rather than the players born in other months [57]. In fact, once a relatively younger player overcomes the selection process and is recruited to play in a high-level club, the chances to become professional increase [55]. Further, relatively younger professional athletes play more matches [58] and have better performances [59–61], higher salaries [62], and longer careers [58–63], which can be explained by a psychological benefit of the relatively younger competing against the relatively older an bigger peers [59], the “underdog hypothesis” [64, 65] and the superior ability [59] and genuine talent of the relatively younger who draw the scouts’ attention to be selected [55].

One limitation of this study is that we examined RAE in a county with a specific sport organization, which is not necessarily a reflection of other places. Nevertheless, sports often are organized in a similar way within Spain and other European countries. In addition, we compared the birth dates of the athletes to the total birth dates in the same county, which does not account for immigrants and emigrants that may alter the distribution of birth dates. However, the impact of this should be low. Thirdly, it is possible that a number of children participated in ballet, dance, and other non-competitive sports in private gyms and would not be included in the present study. Therefore, the conclusions of this study must be considered with this in mind.

## Conclusion

The RAE is present and is a potential problem in Bizkaia, Spain, but the sports in this county are organized in a way that creates very small age-effect sizes, demonstrating that most sports are inclusive for both boys and girls. This comprehensive overview of the phenomenon has allowed us to understand that the potential mechanisms for RAE are multifactorial and complex. In this sense, RAE occurs in sporting contexts with particular characteristics: highly popular sports, team sports, those sports in which body size and physicality are relevant for performance and sports with tight selection processes (both external and internal). In addition, the influence of the parents may be involved and should be taken into account.

## Supporting information

S1 Table Number of male participants divided by sport and birth-year. (DOCX)

S2 Table Number of female participants divided by sport and birth-year. (DOCX)

S3 Table Descriptive statistics of the birth dates of male 9-year-old participants and the general population. (DOCX)

S4 Table Descriptive statistics of the birth dates of male 10-year-old participants and the general population. (DOCX)

S5 Table Descriptive statistics of the birth dates of male 11-year-old participants and the general population. (DOCX)

S6 Table Descriptive statistics of the birth dates of male 12-year-old participants and the general population. (DOCX)

S7 Table Descriptive statistics of the birth dates of male 13-year-old participants and the general population. (DOCX)

S8 Table Descriptive statistics of the birth dates of male 14-year-old participants and the general population. (DOCX)

S9 Table Descriptive statistics of the birth dates of female 9-year-old participants and the general population. (DOCX)

S10 Table Descriptive statistics of the birth dates of female 10-year-old participants and the general population. (DOCX)

S11 Table Descriptive statistics of the birth dates of female 11-year-old participants and the general population. (DOCX)

S12 Table Descriptive statistics of the birth dates of female 12-year-old participants and the general population. (DOCX)

S13 Table Descriptive statistics of the birth dates of female 13-year-old participants and the general population. (DOCX)

S14 Table Descriptive statistics of the birth dates of female 14-year-old participants and the general population. (DOCX)
