# Supplementary material for: The relative age effect in young athletes: A countywide analysis of 9–14-year-old participants in all competitive sports
Source: PLoS One. 2021 Jul 16;16(7):e0254687. doi: 10.1371/journal.pone.0254687 (PMC8284647; doi:10.1371/journal.pone.0254687)
Supplement: S1 Table — (DOCX) [file pone.0254687.s001.docx]

**S1 Table.** Number of male participants divided by sport and birth-year.

|  | **9 y** | **10 y** | **11 y** | **12 y** | **13 y** | **14 y** | **Total** |
| --- | --- | --- | --- | --- | --- | --- | --- |
| Football (all) | 2233 | 2608 | 2671 | 2506 | 2308 | 2112 | 14438 |
| Indoor | 642 | 547 | 313 | 237 | 164 | 704 | 2038 |
| 3^rd^ league | 1330 | 1567 | 1805 | 1499 | 1231 | 135 | 8293 |
| 2^nd^ league | 261 | 494 | 553 | 490 | 546 | 861 | 3048 |
| 1^st^ league |  |  |  | 280 | 367 | 412 | 1059 |
| Basketball (all) | 208 | 292 | 335 | 368 | 345 | 297 | 1845 |
| 2^nd^ league |  |  |  | 319 | 283 | 223 | 825 |
| 1^st^ league |  |  |  | 49 | 62 | 74 | 185 |
| Athletics | 213 | 222 | 221 | 200 | 130 | 163 | 1149 |
| Basque pelota | 156 | 209 | 188 | 172 | 121 | 118 | 964 |
| Trad sport | 220 | 233 | 160 | 131 | 31 | 30 | 805 |
| Taekwondo | 126 | 143 | 135 | 123 | 111 | 79 | 717 |
| Chess | 166 | 158 | 158 | 130 | 44 | 22 | 678 |
| Swimming | 114 | 126 | 114 | 99 | 111 | 86 | 650 |
| Handball | 80 | 103 | 98 | 111 | 141 | 105 | 638 |
| Karate | 100 | 72 | 71 | 70 | 63 | 48 | 424 |
| Hockey | 62 | 76 | 58 | 48 | 52 | 50 | 346 |
| Cycling | 35 | 55 | 72 | 56 | 58 | 52 | 328 |
| Judo | 54 | 68 | 64 | 45 | 39 | 25 | 295 |
| Rugby | 40 | 35 | 33 | 43 | 36 | 78 | 265 |
| Multisport | 126 | 103 | 4 | 2 |  |  | 235 |
| Water polo | 14 | 44 | 36 | 34 | 23 | 23 | 174 |
| Tennis | 11 | 37 | 26 | 19 | 32 | 26 | 151 |
| Rowing | 3 | 9 | 9 | 19 | 36 | 73 | 149 |
| Triathlon | 14 | 22 | 21 | 16 | 31 | 25 | 129 |
| Padel | 4 | 10 | 21 | 14 | 19 | 19 | 87 |
| Baseball | 16 | 24 | 17 | 25 | 4 |  | 86 |
| Table tennis | 6 | 5 | 14 | 6 | 18 | 24 | 73 |
| Gymnastics | 17 | 18 | 3 | 11 | 11 | 12 | 72 |
| Volleyball | 13 | 18 | 9 | 5 | 2 | 13 | 60 |
| Total | 4031 | 4690 | 4538 | 4253 | 3766 | 3480 | 24758 |

Trad sport: traditional sport; y: years old
