# Supplementary material for: The relative age effect in young athletes: A countywide analysis of 9–14-year-old participants in all competitive sports
Source: PLoS One. 2021 Jul 16;16(7):e0254687. doi: 10.1371/journal.pone.0254687 (PMC8284647; doi:10.1371/journal.pone.0254687)
Supplement: S2 Table — (DOCX) [file pone.0254687.s002.docx]

**S2 Table.** Number of female participants divided by sport and birth-year.

|  | **9 y** | **10 y** | **11 y** | **12 y** | **13 y** | **14 y** | **Total** |
| --- | --- | --- | --- | --- | --- | --- | --- |
| Basketball (all) | 392 | 725 | 711 | 780 | 668 | 504 | 3780 |
| 2^nd^ league | 392 | 725 | 711 | 713 | 589 | 404 | 3534 |
| 1st league |  |  |  | 67 | 79 | 100 | 246 |
| Rhythmic Gym | 322 | 311 | 261 | 234 | 175 | 131 | 1434 |
| Handball | 122 | 229 | 245 | 275 | 258 | 178 | 1307 |
| Football | 154 | 167 | 252 | 248 | 232 | 209 | 1262 |
| Outdoor | 83 | 126 | 224 | 225 | 221 | 205 | 1084 |
| Indoor | 71 | 41 | 28 | 23 | 11 | 4 | 178 |
| Athletics | 183 | 238 | 225 | 192 | 174 | 167 | 1179 |
| Volleyball | 77 | 87 | 141 | 174 | 93 | 162 | 734 |
| Swimming | 106 | 120 | 154 | 146 | 59 | 51 | 636 |
| Trad sport | 145 | 158 | 130 | 121 | 18 | 18 | 590 |
| Taekwondo | 81 | 83 | 77 | 77 | 63 | 57 | 438 |
| Chess | 82 | 59 | 56 | 33 | 15 | 4 | 249 |
| Hockey | 59 | 38 | 29 | 44 | 15 | 22 | 207 |
| Karate | 27 | 34 | 37 | 51 | 20 | 21 | 190 |
| Artistic skating | 37 | 33 | 40 | 20 | 21 | 29 | 180 |
| Basque pelota | 18 | 25 | 37 | 26 | 23 | 8 | 137 |
| Skate-racing | 22 | 30 | 29 | 19 | 12 |  | 112 |
| Aerobic | 8 | 19 | 24 | 12 | 24 | 24 | 111 |
| Multisport | 59 | 22 | 14 | 2 |  |  | 97 |
| Judo | 22 | 16 | 23 | 13 | 14 | 7 | 95 |
| Padel | 6 | 21 | 13 | 16 | 14 | 22 | 92 |
| Tennis | 9 | 13 | 14 | 15 | 15 | 22 | 88 |
| Triathlon | 8 | 7 | 18 | 16 | 22 | 15 | 86 |
| Baseball | 15 | 12 | 18 | 27 |  |  | 72 |
| Synchronized swimming | 11 | 9 | 28 | 13 | 8 | 3 | 72 |
| Cycling | 4 | 18 | 15 | 17 | 9 | 6 | 69 |
| Water polo | 9 | 7 | 6 | 13 | 6 | 9 | 50 |
| Rugby | 8 | 7 | 5 | 6 | 12 | 7 | 45 |
| Rowing |  |  | 5 | 8 | 11 | 5 | 29 |
| Canoeing |  | 1 | 8 | 3 | 6 | 9 | 27 |
| Artistic gym | 4 | 4 | 3 | 8 | 4 | 3 | 26 |
| Climbing | 1 | 4 | 2 | 5 | 4 | 5 | 21 |
| Archery | 1 | 1 | 3 | 4 | 4 | 3 | 16 |
| Skiing | 2 |  |  |  |  | 11 | 13 |
| Trampolining |  | 3 |  | 2 | 4 | 1 | 10 |
| Table tennis |  | 1 | 2 | 3 | 1 | 2 | 9 |
| Total (n) | 1994 | 2502 | 2625 | 2623 | 2004 | 1715 | 13463 |

Gymn: gymnastics; Trad sport: traditional sport; y: years old
