# Supplementary material for: The relative age effect in young athletes: A countywide analysis of 9–14-year-old participants in all competitive sports
Source: PLoS One. 2021 Jul 16;16(7):e0254687. doi: 10.1371/journal.pone.0254687 (PMC8284647; doi:10.1371/journal.pone.0254687)
Supplement: S3 Table — (DOCX) [file pone.0254687.s003.docx]

**S3 Table.** Descriptive statistics of the birth dates of male 9-year-old participants and the general population.

|  | **Total (n)** | **Q1** | **Q2** | **Q3** | **Q4** | **Median** | **IQR** |
| --- | --- | --- | --- | --- | --- | --- | --- |
| Football (all) | 2233 | 25.8% | 27.9% | 24.0% | 22.4% | 195.0 | 103.00-277.0 |
| Part | 1330 | 25.1% | 28.4% | 23.4% | 23.1% | 195.0 | 98.00-275.00 |
| Comp | 261 | 34.9% | 32.2% | 21.8% | 11.1% | 240.0 | 151.50-315.00 |
| Indoor | 642 | 23.4% | 24.9% | 26.2% | 25.5% | 172.0 | 89.00-268.25 |
| Trad sport | 220 | 21.4% | 29.1% | 26.8% | 22.7% | 186.0 | 108-25-263.25 |
| Athletics | 213 | 21.6% | 27.7% | 24.9% | 25.8% | 181.0 | 87.00-263.00 |
| Basketball | 208 | 28.8% | 26.0% | 22.6% | 22.6% | 198.0 | 113.25-289.75 |
| Chess | 166 | 25.9% | 22.9% | 25.3% | 25.9% | 165.0 | 89.50-277.25 |
| Basque pelota | 156 | 27.6% | 30.8% | 19.2% | 22.4% | 202.0 | 111.75-292.50 |
| Taekwondo | 126 | 25.4% | 25.4% | 26.2% | 23.0% | 184.0 | 99.50-276.25 |
| Multisport | 126 | 24.6% | 28.6% | 23.0% | 23.8% | 193.0 | 93.75-270.00 |
| Swimming | 114 | 28.1% | 27.2% | 25.4% | 19.3% | 205.5 | 108.75-286.75 |
| Karate | 100 | 31.0% | 28.0% | 25.0% | 16.0% | 222.0 | 119.25-291.75 |
| Hockey | 62 | 17.7% | 29.0% | 27.4% | 25.8% | 165.5 | 83.25-250.25 |
| Handball | 80 | 22.5% | 23.8% | 30.0% | 23.8% | 165.5 | 93.50-263.00 |
| Judo | 54 | 22.2% | 25.9% | 29.6% | 22.2% | 174.5 | 106.25-263.25 |
| Rugby | 40 | 20.0% | 30.0% | 30.0% | 20.0% | 174.0 | 104.75-260.25 |
| Cycling | 35 | 31.4% | 14.3% | 31.4% | 22.9% | 177.0 | 92.00-294.00 |
| Volleyball | 13 | 38.5% | 23.1% | 23.1% | 15.4% | 193.0 | 100.5-298.00 |
| Gymnastics | 17 | 23.5% | 29.4% | 29.4% | 17.6% | 213.0 | 93.00-288.00 |
| Baseball | 16 | 43.8% | 18.8% | 12.5% | 25.0% | 207.0 | 52.00-325.75 |
| Triathlon | 14 | 21.4% | 35.7% | 14.3% | 28.6% | 241.5 | 82.00-272.00 |
| Water polo | 14 | 28.6% | 28.6% | 14.3% | 28.6% | 222.5 | 82.00-298.75 |
| Tennis | 11 | 36.4% | 18.2% | 18.2% | 27.3% | 185.0 | 24.00-312.00 |
| Table tennis | 6 | 16.7% |  | 50.0% | 33.3% | 125.5 | 53.75-207.25 |
| Padel | 4 | 25.0% | 25.0% | 25.0% | 25.0% | 181.5 | 74.25-323.25 |
| Rowing | 3 | 66.7% |  |  | 33.3% | 281.0 |  |
| Total |  | 25.6% | 27.3% | 24.4% | 22.7% | 193.00 | 277.00-101.00 |
| Total (n) | 4031 | 1031 | 1103 | 983 | 914 |  |  |
| Gen pop (n) | 5171 | 1260 | 1332 | 1268 | 1311 |  |  |

n: number of players; Q: birth quarter; IQR: interquartile range (25^th^ and 75^th^ percentiles are shown); Part: participation; Comp: competition; Perf: performance; Trad: traditional; Gen pop: general population
