# Supplementary material for: The relative age effect in young athletes: A countywide analysis of 9–14-year-old participants in all competitive sports
Source: PLoS One. 2021 Jul 16;16(7):e0254687. doi: 10.1371/journal.pone.0254687 (PMC8284647; doi:10.1371/journal.pone.0254687)
Supplement: S4 Table — (DOCX) [file pone.0254687.s004.docx]

**S4 Table.** Descriptive statistics of the birth dates of male 10-year-old participants and the general population.

|  | **Total (n)** | **Q1** | **Q2** | **Q3** | **Q4** | **Median** | **IQR** |
| --- | --- | --- | --- | --- | --- | --- | --- |
| Football (all) | 2608 | 25.5% | 27.4% | 24.3% | 22.8% | 194.00 | 101.00-276.75 |
| Part | 1567 | 24.0% | 26.7% | 25.3% | 24% | 186.00 | 95.00-271.00 |
| Comp | 494 | 30.6% | 30.0% | 25.3% | 17.6% | 215.00 | 123.75-290.00 |
| Indoor | 547 | 25.4% | 27.1% | 23.6% | 23.9% | 191.00 | 95.00-277.00 |
| Basketball | 292 | 22.6% | 26.7% | 25.3% | 25.3% | 180.50 | 90.25-267.75 |
| Trad sport | 233 | 27.5% | 24.5% | 21.5% | 26.6% | 186.00 | 85.50-288.50 |
| Athletics | 222 | 24.8% | 31.1% | 21.2% | 23.0% | 201.00 | 93.00-275.50 |
| Basque pelota | 209 | 25.8% | 21.1% | 30.6% | 22.5% | 174.00 | 96.00-279.00 |
| Chess | 158 | 24.1% | 24.7% | 29.7% | 21.5% | 178.50 | 106.75-270.75 |
| Taekwondo | 143 | 24.5% | 30.8% | 22.4% | 22.4% | 204.00 | 95.00-274.00 |
| Swimming | 126 | 24.6% | 29.4% | 27.8% | 18.3% | 191.00 | 108.00-274.00 |
| Handball | 103 | 23.3% | 24.3% | 24.3% | 28.2% | 173.00 | 74.00-268.00 |
| Multisport | 103 | 29.1% | 24.3% | 21.4% | 25.2% | 189.00 | 86.00-284.00 |
| Hockey | 76 | 27.6% | 17.1% | 31.6% | 23.7% | 170.50 | 95.00-291.50 |
| Karate | 72 | 26.4% | 30.6% | 19.4% | 23.6% | 203.50 | 99.00-279.00 |
| Judo | 68 | 27.9% | 26.5% | 23.5% | 22.1% | 201.00 | 104.50-282.50 |
| Cycling | 55 | 25.5% | 10.9% | 30.9% | 32.7% | 143.00 | 80.00-276.00 |
| Water polo | 44 | 27.3% | 25.0% | 20.5% | 27.3% | 194.50 | 63.25-292.00 |
| Tennis | 37 | 35.1% | 21.6% | 18.9% | 24.3% | 212.00 | 87.50-298.50 |
| Rugby | 35 | 17.1% | 37.1% | 31.4% | 14.3% | 206.00 | 128.00-269.00 |
| Baseball | 24 | 29.2% | 16.7% | 33.3% | 20.8% | 171.50 | 108.75-275.00 |
| Triathlon | 22 | 27.3% | 36.4% | 27.3% | 9.1% | 210.50 | 157.50-291.75 |
| Gymnastics | 18 | 11.1% | 27.8% | 27.8% | 33.3% | 151.00 | 55.25-233.25 |
| Volleyball | 18 | 33.3% | 16.7% | 22.2% | 27.8% | 208.50 | 81.25-299.75 |
| Padel | 10 | 50.0% | 20.0% |  | 30.0% | 268.50 | 46.00-323.00 |
| Rowing | 9 | 33.3% |  | 44.4% | 22.2% | 164.00 | 108.00-330.00 |
| Table tennis | 5 | 40.0% |  | 60.0% |  | 162.00 | 137.00-277.50 |
| Total |  | 25.5% | 26.5% | 24.7% | 23.2% | 191.00 | 99.00-277.00 |
| Total (n) | 4690 | 1198 | 1245 | 1158 | 1089 |  |  |
| Gen pop (n) | 5153 | 1216 | 1312 | 1338 | 1287 |  |  |

n: number of players; Q: birth quarter; IQR: interquartile range (25^th^ and 75^th^ percentiles are shown); Part: participation; Comp: competition; Perf: performance; Trad.: traditional; Gen pop: general population
