# Supplementary material for: The relative age effect in young athletes: A countywide analysis of 9–14-year-old participants in all competitive sports
Source: PLoS One. 2021 Jul 16;16(7):e0254687. doi: 10.1371/journal.pone.0254687 (PMC8284647; doi:10.1371/journal.pone.0254687)
Supplement: S5 Table — (DOCX) [file pone.0254687.s005.docx]

**S5 Table.** Descriptive statistics of the birth dates of male 11-year-old participants and the general population.

|  | **Total (n)** | **Q1** | **Q2** | **Q3** | **Q4** | **Median** | **IQR** |
| --- | --- | --- | --- | --- | --- | --- | --- |
| Football (all) | 2671 | 25.5% | 25.9% | 24.1% | 24.5% | 189.00 | 94.00-277.00 |
| Part | 1805 | 25.3% | 24.0% | 24.7% | 26.1% | 181.00 | 86.50-276.00 |
| Comp | 553 | 27.5% | 31.1% | 21.9% | 19.5% | 210.00 | 116.50-283.00 |
| Indoor | 313 | 23.6% | 27.8% | 24.6% | 24.0% | 186.00 | 95.00-267.00 |
| Basketball | 335 | 25.7% | 27.8% | 23.9% | 22.7% | 194.00 | 101.00-280.00 |
| Athletics | 221 | 23.1% | 22.6% | 33.5% | 20.8% | 169.00 | 98.50-270.00 |
| Basque pelota | 188 | 29.3% | 23.9% | 29.3% | 17.6% | 197.50 | 112.00-293.25 |
| Trad sport | 160 | 22.5% | 28.1% | 23.1% | 26.3% | 187.50 | 89.25-261.75 |
| Chess | 158 | 27.8% | 24.1% | 24.7% | 23.4% | 189.50 | 95.00-288.00 |
| Taekwondo | 135 | 18.5% | 32.6% | 19.3% | 29.6% | 187.00 | 65.00-259.00 |
| Swimming | 114 | 24.6% | 19.3% | 32.5% | 23.7% | 174.50 | 97.50-271.25 |
| Handball | 98 | 22.4% | 31.6% | 17.3% | 28.6% | 197.00 | 90.75-266.00 |
| Cycling | 72 | 20.8% | 23.6% | 27.8% | 27.8% | 169.00 | 77.25-266.00 |
| Karate | 71 | 28.2% | 18.3% | 25.4% | 28.2% | 175.00 | 79.00-296.00 |
| Judo | 64 | 31.3% | 25.0% | 21.9% | 21.9% | 200.50 | 99.25-286.50 |
| Hockey | 58 | 24.1% | 25.9% | 17.2% | 32.8% | 181.50 | 62.25-269.75 |
| Water polo | 36 | 13.9% | 30.6% | 33.3% | 22.2% | 165.50 | 101.25-226.25 |
| Rugby | 33 | 21.2% | 42.4% | 27.3% | 9.1% | 224.00 | 146.50-264.00 |
| Tennis | 26 | 30.8% | 19.2% | 26.9% | 23.1% | 177.00 | 112.00-284.75 |
| Padel | 21 | 23.8% | 19.0% | 19.0% | 38.1% | 155.00 | 62.50-274.00 |
| Triathlon | 21 | 19.0% | 23.8% | 38.1% | 19.0% | 176.00 | 98.50-265.50 |
| Baseball | 17 | 11.8% | 23.5% | 17.6% | 47.1% | 129.00 | 44.00-254.00 |
| Table tennis | 14 | 28.6% | 21.4% | 14.3% | 35.7% | 187.00 | 36.50-287.50 |
| Volleyball | 9 | 33.3% | 33.3% | 22.2% | 11.1% | 224.00 | 156.00-306.50 |
| Rowing | 9 | 44.4% |  | 11.1% | 44.4% | 118.00 | 25.50-353.00 |
| Multisport | 4 | 75.0% | 25.0% |  |  | 283.50 | 257.75-328.00 |
| Gymnastics | 3 | 66.7% |  | 33.3% |  | 303.00 |  |
| Total |  | 25.2% | 25.8% | 24.7% | 24.3% | 188.00 | 95.00-275.00 |
| Total (n) | 4538 | 1145 | 1171 | 1119 | 1103 |  |  |
| Gen pop (n) | 5080 | 1208 | 1305 | 1296 | 1271 |  |  |

n: number of players; Q: birth quarter; IQR: interquartile range (25^th^ and 75^th^ percentiles are shown); Part: participation; Comp: competition; Perf: performance; Trad: traditional; Gen pop: general population
