# Supplementary material for: The relative age effect in young athletes: A countywide analysis of 9–14-year-old participants in all competitive sports
Source: PLoS One. 2021 Jul 16;16(7):e0254687. doi: 10.1371/journal.pone.0254687 (PMC8284647; doi:10.1371/journal.pone.0254687)
Supplement: S6 Table — (DOCX) [file pone.0254687.s006.docx]

**S6 Table.** Descriptive statistics of the birth dates of male 12-year-old participants and the general population.

|  | **Total (n)** | **Q1** | **Q2** | **Q3** | **Q4** | **Median** | **IQR** |
| --- | --- | --- | --- | --- | --- | --- | --- |
| Football (all) | 2506 | 25.7% | 26.4% | 23.4% | 24.5% | 193.00 | 93.00-277.00 |
| Part | 1499 | 23.0% | 25.9% | 24.9% | 26.2% | 180.00 | 88.00-267.00 |
| Comp | 490 | 25.3% | 27.8% | 24.3% | 22.7% | 193.50 | 102.25-276.50 |
| Perf | 280 | 42.5% | 26.1% | 15.4% | 16.1% | 254.50 | 148.50-314.00 |
| Indoor | 237 | 23.6% | 27.4% | 21.9% | 27.0% | 187.00 | 83.50-272.00 |
| Basketball (all) | 368 | 27.2% | 24.2% | 27.4% | 21.2% | 190.00 | 100.25-284.75 |
| Comp | 319 | 26.6% | 24.1% | 27.6% | 21.6% | 189.00 | 98.00-281.00 |
| Perf | 49 | 30.6% | 24.5% | 26.5% | 18.4% | 200.00 | 126.00-301.00 |
| Athletics | 200 | 23.0% | 26.0% | 25.0% | 26.0% | 178.50 | 88.25-266.75 |
| Basque pelota | 172 | 25.6% | 20.3% | 30.8% | 23.3% | 173.50 | 100.50-277.25 |
| Trad sport | 131 | 29.0% | 19.8% | 16.0% | 35.1% | 177.00 | 46.00-285.00 |
| Chess | 130 | 26.2% | 24.6% | 26.9% | 22.3% | 184.50 | 95.75-279.00 |
| Taekwondo | 123 | 17.1% | 24.4% | 25.2% | 33.3% | 157.00 | 72.00-243.00 |
| Handball | 111 | 27.9% | 27.0% | 22.5% | 22.5% | 196.00 | 108.00-290.00 |
| Swimming | 99 | 24.2% | 27.3% | 31.3% | 17.2% | 194.00 | 113.00-274.00 |
| Karate | 70 | 30.0% | 21.4% | 28.6% | 20.0% | 194.00 | 105.25-293.50 |
| Cycling | 56 | 28.6% | 23.2% | 25.0% | 23.2% | 192.00 | 95.75-293.50 |
| Hockey | 48 | 18.8% | 22.9% | 31.3% | 27.1% | 144.50 | 82.25-258.75 |
| Judo | 45 | 20.0% | 24.4% | 33.3% | 22.2% | 166.00 | 99.50-256.50 |
| Rugby | 43 | 11.6% | 32.6% | 27.9% | 27.9% | 158.00 | 76.00-230.00 |
| Water polo | 34 | 14.7% | 41.2% | 20.6% | 23.5% | 200.00 | 86.25-261.75 |
| Baseball | 25 | 16.0% | 32.0% | 32.0% | 20.0% | 157.00 | 116.50-232.50 |
| Rowing | 19 | 15.8% | 42.1% | 10.5% | 31.6% | 196.00 | 80.00-274.00 |
| Tennis | 19 | 26.3% | 31.6% | 21.1% | 21.1% | 217.00 | 137.00-286.00 |
| Triathlon | 16 | 18.8% | 12.5% | 37.5% | 31.3% | 153.50 | 86.50-253.75 |
| Padel | 14 | 14.3% | 28.6% | 50.0% | 7.1% | 166.50 | 115.25-272.25 |
| Gymnastics | 11 | 18.2% | 18.2% | 27.3% | 36.4% | 107.00 | 72.00-259.00 |
| Table tennis | 6 | 16.7% |  | 50.0% | 33.3% | 123.50 | 18.75-196.25 |
| Volleyball | 5 | 20.0% |  | 40.0% | 40.0% | 154.00 | 75.00-222.50 |
| Multisport | 2 | 50.0% |  | 50.0% |  | 214.00 |  |
| Total |  | 25.1% | 25.7% | 24.8% | 24.5% | 187.00 | 93.00-275.00 |
| Total (n) | 4253 | 1069 | 1091 | 1053 | 1040 |  |  |
| Gen pop (n) | 4783 | 1132 | 1211 | 1206 | 1234 |  |  |

n: number of players; Q: birth quarter; IQR: interquartile range (25^th^ and 75^th^ percentiles are shown); Part: participation; Comp: competition; Perf: performance; Trad: traditional; Gen pop: general population
