# Supplementary material for: The relative age effect in young athletes: A countywide analysis of 9–14-year-old participants in all competitive sports
Source: PLoS One. 2021 Jul 16;16(7):e0254687. doi: 10.1371/journal.pone.0254687 (PMC8284647; doi:10.1371/journal.pone.0254687)
Supplement: S7 Table — (DOCX) [file pone.0254687.s007.docx]

**S7 Table.** Descriptive statistics of the birth dates of male 13-year-old participants and the general population.

|  | **Total (n)** | **Q1** | **Q2** | **Q3** | **Q4** | **Median** | **IQR** |
| --- | --- | --- | --- | --- | --- | --- | --- |
| Football (all) | 2308 | 25.6% | 25.4% | 24.9% | 24.0% | 188.00 | 97.00-277.00 |
| Part | 1231 | 23.6% | 24.0% | 26.1% | 26.2% | 174.00 | 88.00-270.00 |
| Comp | 546 | 25.5% | 27.5% | 23.8% | 23.3% | 199.00 | 99.75-276.25 |
| Perf | 367 | 32.7% | 28.9% | 20.7% | 17.7% | 221.00 | 132.00-293.00 |
| Indoor | 164 | 25.6% | 21.3% | 29.3% | 23.8% | 160.50 | 94.00-280.00 |
| Basketball (all) | 345 | 23.5% | 26.1% | 24.3% | 26.1% | 178.00 | 87.00-269.00 |
| Comp | 283 | 21.6% | 26.5% | 25.8% | 26.1% | 174.00 | 87.00-267.00 |
| Perf | 62 | 32.3% | 24.2% | 17.7% | 25.8% | 212.00 | 83.75-307.50 |
| Handball | 141 | 22.0% | 27.7% | 24.8% | 25.5% | 182.00 | 86.50-268.00 |
| Athletics | 130 | 28.5% | 22.3% | 22.3% | 26.9% | 187.00 | 82.75-286.50 |
| Basque pelota | 121 | 28.1% | 22.3% | 25.6% | 24.0% | 184.00 | 95.50-280.50 |
| Swimming | 111 | 24.3% | 25.2% | 23.4% | 27.0% | 183.00 | 82.00-274.00 |
| Taekwondo | 111 | 21.6% | 28.8% | 22.5% | 27.0% | 184.00 | 81.00-260.00 |
| Karate | 63 | 31.7% | 17.5% | 22.2% | 28.6% | 173.00 | 77.00-310.00 |
| Cycling | 58 | 32.8% | 24.1% | 19.0% | 24.1% | 214.50 | 91.25-303-00 |
| Hockey | 52 | 34.6% | 23.1% | 13.5% | 28.8% | 220.50 | 81.75-292.50 |
| Chess | 44 | 15.9% | 13.6% | 43.2% | 27.3% | 161.00 | 87.25-218.50 |
| Judo | 39 | 25.6% | 28.2% | 28.2% | 17.9% | 198.00 | 130.00-294.00 |
| Rowing | 36 | 27.8% | 22.2% | 27.8% | 22.2% | 192.00 | 103.25-280.25 |
| Rugby | 36 | 19.4% | 25.0% | 19.4% | 36.1% | 149.00 | 61.25-269.75 |
| Tennis | 32 | 31.3% | 25.0% | 21.9% | 21.9% | 212.50 | 156.75-289.50 |
| Trad. sport | 31 | 25.8% | 16.1% | 32.3% | 25.8% | 159.00 | 91.00-278.00 |
| Triathlon | 31 | 19.4% | 19.4% | 25.8% | 35.5% | 144.00 | 66.00-268.00 |
| Water polo | 23 | 30.4% | 26.1% | 26.1% | 17.4% | 202.00 | 125.00-307.00 |
| Padel | 19 | 26.3% | 31.6% | 15.8% | 26.3% | 220.00 | 63.00-279.00 |
| Table tennis | 18 | 44.4% | 22.2% | 27.8% | 5.6% | 243.00 | 161.25-318.25 |
| Gymnastics | 11 | 27.3% | 27.3% | 27.3% | 18.2% | 220.00 | 125.00-307.00 |
| Baseball | 4 | 25.0% |  | 50.0% | 25.0% | 136.50 | 54.00-276.75 |
| Volleyball | 2 |  | 50.0% |  | 50.0% | 130.00 |  |
| Total |  | 25.6% | 25.0% | 24.6% | 24.7% | 187.00 | 93.00-276.00 |
| Total (n) | 3766 | 965 | 942 | 928 | 931 |  |  |
| Gen pop (n) | 4722 | 1123 | 1205 | 1194 | 1200 |  |  |

n: number of players; Q: birth quarter; IQR: interquartile range (25^th^ and 75^th^ percentiles are shown)
