# Supplementary material for: The relative age effect in young athletes: A countywide analysis of 9–14-year-old participants in all competitive sports
Source: PLoS One. 2021 Jul 16;16(7):e0254687. doi: 10.1371/journal.pone.0254687 (PMC8284647; doi:10.1371/journal.pone.0254687)
Supplement: S8 Table — (DOCX) [file pone.0254687.s008.docx]

**S8 Table.** Descriptive statistics of the birth dates of male 14-year-old participants and the general population.

|  | **Total (n)** | **Q1** | **Q2** | **Q3** | **Q4** | **Median** | **IQR** |
| --- | --- | --- | --- | --- | --- | --- | --- |
| Football (all) | 2112 | 27.2% | 26.2% | 23.6% | 23.0% | 196.50 | 99.00-284.00 |
| Part | 861 | 22.9% | 24.9% | 24.5% | 27.8% | 173.00 | 81.50-266.00 |
| Comp | 704 | 27.8% | 26.4% | 23.4% | 22.3% | 204.00 | 101.25-286.00 |
| Perf | 412 | 35.0% | 29.6% | 20.6% | 14.8% | 230.00 | 138.00-299.00 |
| Indoor | 135 | 28.1% | 23.7% | 27.4% | 20.7% | 190.00 | 103.00-297.00 |
| Basketball (all) | 297 | 28.6% | 28.3% | 20.9% | 22.2% | 206.00 | 100.50-284.00 |
| Comp | 223 | 29.1% | 28.3% | 20.2% | 22.4% | 207.00 | 100.00-284.00 |
| Perf | 74 | 27.0% | 28.4% | 23.0% | 21.6% | 200.00 | 100.75-282.00 |
| Athletics | 163 | 29.4% | 27.0% | 22.1% | 21.5% | 211.00 | 104.00-284.00 |
| Basque pelota | 118 | 24.6% | 20.3% | 30.5% | 24.6% | 157.50 | 92.00-276.00 |
| Handball | 105 | 29.5% | 25.7% | 24.8% | 20.0% | 208.00 | 124.00-297.00 |
| Swimming | 86 | 27.9% | 24.4% | 23.3% | 24.4% | 216.00 | 91.75-283.25 |
| Taekwondo | 79 | 25.3% | 11.4% | 24.1% | 39.2% | 123.00 | 45.00-277.00 |
| Rugby | 78 | 29.5% | 17.9% | 32.1% | 20.5% | 176.50 | 100.00-294.00 |
| Rowing | 73 | 23.3% | 34.2% | 28.8% | 13.7% | 210.00 | 117.00-267.50 |
| Cycling | 52 | 25.0% | 23.1% | 28.8% | 23.1% | 173.50 | 96.75-282.00 |
| Hockey | 50 | 30.0% | 28.0% | 20.0% | 22.0% | 210.50 | 101.25-295.00 |
| Karate | 48 | 22.9% | 16.7% | 35.4% | 25.0% | 168.00 | 75.25-263.25 |
| Trad sport | 30 | 30.0% | 20.0% | 20.0% | 30.0% | 173.00 | 81.00-312.00 |
| Tennis | 26 | 34.6% | 15.4% | 19.2% | 30.8% | 181.50 | 88.75-302.75 |
| Judo | 25 | 24.0% | 24.0% | 36.0% | 16.0% | 178.00 | 98.50-272.00 |
| Triathlon | 25 | 12.0% | 32.0% | 28.0% | 28.0% | 172.00 | 76.00-222.00 |
| Table tennis | 24 | 37.5% | 25.0% | 16.7% | 20.8% | 238.00 | 109.50-298.50 |
| Water polo | 23 | 34.8% | 30.4% | 21.7% | 13.0% | 243.00 | 111.00-293.00 |
| Chess | 22 | 27.3% | 31.8% | 13.6% | 27.3% | 202.50 | 69.50-299.50 |
| Volleyball | 13 | 69.2% |  | 15.4% | 15.4% | 313.00 | 104.00-325.00 |
| Padel | 19 | 15.8% | 15.8% | 42.1% | 26.3% | 139.00 | 91.00-206.00 |
| Gymnastics | 12 | 16.7% | 8.3% | 8.3% | 66.7% | 58.50 | 20.00-215.75 |
| Total |  | 27.4% | 25.4% | 24.0% | 23.2% | 170.0 | 81.0-267.0 |
| Total (n) | 3480 | 955 | 884 | 835 | 806 | 195.00 | 98.00-284.00 |
| Gen pop (n) | 4553 | 1172 | 1173 | 1093 | 1115 |  |  |

n: number of players; Q: birth quarter; IQR: interquartile range (25^th^ and 75^th^ percentiles are shown); Part: participation; Comp: competition; Perf: performance; Trad: traditional; Gen pop: general population
