# Supplementary material for: The relative age effect in young athletes: A countywide analysis of 9–14-year-old participants in all competitive sports
Source: PLoS One. 2021 Jul 16;16(7):e0254687. doi: 10.1371/journal.pone.0254687 (PMC8284647; doi:10.1371/journal.pone.0254687)
Supplement: S9 Table — (DOCX) [file pone.0254687.s009.docx]

**S9 Table.** Descriptive statistics of the birth dates of female 9-year-old participants and the general population.

|  | **Total (n)** | **Q1** | **Q2** | **Q3** | **Q4** | **Median** | **IQR** |
| --- | --- | --- | --- | --- | --- | --- | --- |
| Basketball | 392 | 25.3% | 25.5% | 24.7% | 24.5% | 186.50 | 95.00-275.00 |
| Rhythmic Gym | 322 | 25.5% | 25.2% | 25.5% | 23.9% | 188.00 | 98.00-279.25 |
| Athletics | 183 | 29.0% | 20.2% | 24.0% | 26.8% | 181.00 | 79.00-289.00 |
| Football | 154 | 31.2% | 21.4% | 22.7% | 24.7% | 190.00 | 94.50-292.25 |
| Trad sport | 145 | 24.8% | 27.6% | 21.4% | 26.2% | 197.00 | 82.00-273.50 |
| Handball | 122 | 23.0% | 25.4% | 32.0% | 19.7% | 173.00 | 107.00-253.25 |
| Swimming | 106 | 29.2% | 19.8% | 27.4% | 23.6% | 177.00 | 96.00-289.25 |
| Chess | 82 | 24.4% | 24.4% | 19.5% | 31.7% | 178.00 | 63.25-273.75 |
| Taekwondo | 81 | 24.7% | 18.5% | 23.5% | 33.3% | 154.00 | 74.50-271.50 |
| Volleyball | 77 | 13.0% | 32.5% | 27.3% | 27.3% | 154.00 | 65.00-242.00 |
| Hockey | 59 | 22.0% | 37.3% | 18.6% | 22.0% | 209.00 | 95.00-269.00 |
| Multisport | 59 | 25.4% | 20.3% | 20.3% | 33.9% | 164.00 | 50.00-279.00 |
| Artistic skating | 37 | 21.6% | 32.4% | 16.2% | 29.7% | 191.00 | 64.50-262.00 |
| Karate | 27 | 37.0% | 25.9% | 25.9% | 11.1% | 236.00 | 133.00-333.0 |
| Judo | 22 | 36.4% | 13.6% | 13.6% | 36.4% | 181.50 | 45.75-304.25 |
| Skate-racing | 22 | 31.8% | 18.2% | 27.3% | 22.7% | 177.50 | 95.00-301.00 |
| Basque pelota | 18 | 50.0% | 16.7% | 16.7% | 16.7% | 273.00 | 116.50-332.50 |
| Baseball | 15 | 13.3% | 40.0% | 20.0% | 26.7% | 187.00 | 47.00-262.00 |
| Synchronized sw | 11 | 36.4% | 18.2% | 27.3% | 18.2% | 205.00 | 131.00-290.00 |
| Tennis | 9 | 11.1% | 22.2% | 33.3% | 33.3% | 126.00 | 64.50-236.00 |
| Water polo | 9 | 44.4% | 22.2% | 11.1% | 22.2% | 215.00 | 85.00-347.00 |
| Aerobic | 8 | 50.0% | 12.5% | 12.5% | 25.0% | 265.50 | 77.50-343.25 |
| Rugby | 8 | 25.0% | 12.5% | 25.0% | 37.5% | 154.00 | 43.00-274.00 |
| Triathlon | 8 | 37.5% | 12.5% | 12.5% | 37.5% | 175.50 | 69.00-315.00 |
| Padel | 6 | 33.3% | 33.3% | 16.7% | 16.7% | 201.50 | 123.25-317.50 |
| Cycling | 4 | 50.0% |  | 25.0% | 25.0% | 220.00 | 78.75-308.75 |
| Artistic Gym | 4 | 25.0% | 25.0% | 25.0% | 25.0% | 180.50 | 50.25-275.50 |
| Skiing | 2 |  | 100.0% |  |  | 235.00 |  |
| Climbing | 1 |  |  |  | 100.0% |  |  |
| Archery | 1 |  |  | 100.0% |  |  |  |
| Total |  | 26.2% | 24.4% | 24.0% | 25.4% | 186.00 | 89.00-279.00 |
| Total (n) | 1994 | 522 | 486 | 479 | 507 |  |  |
| Gen pop (n) | 4950 | 1226 | 1199 | 1231 | 1294 |  |  |

Q: birth quarter; IQR: interquartile range (25^th^ and 75^th^ percentiles are shown); Gym: gymnastics; Trad: traditional; sw: swimming; Gen pop: general population
