# Supplementary material for: The relative age effect in young athletes: A countywide analysis of 9–14-year-old participants in all competitive sports
Source: PLoS One. 2021 Jul 16;16(7):e0254687. doi: 10.1371/journal.pone.0254687 (PMC8284647; doi:10.1371/journal.pone.0254687)
Supplement: S10 Table — (DOCX) [file pone.0254687.s010.docx]

**S10 Table.** Descriptive statistics of the birth dates of female 10-year-old participants and the general population.

|  | **Total (n)** | **Q1** | **Q2** | **Q3** | **Q4** | **Median** | **IQR** |
| --- | --- | --- | --- | --- | --- | --- | --- |
| Basketball | 725 | 28.1% | 27.7% | 22.5% | 21.7% | 206.00 | 105.50-288.00 |
| Rhythmic Gym | 311 | 24.1% | 31.2% | 19.9% | 24.8% | 197.00 | 93.00-273.00 |
| Athletics | 238 | 25.2% | 23.9% | 20.6% | 30.3% | 176.00 | 70.75-275.25 |
| Handball | 229 | 24.0% | 30.6% | 22.3% | 23.1% | 191.00 | 96.50-271.00 |
| Football | 167 | 25.7% | 23.4% | 24.0% | 26.9% | 176.00 | 85.00-278.00 |
| Trad sport | 158 | 20.9% | 28.5% | 24.7% | 25.9% | 182.00 | 89.50-263.25 |
| Swimming | 120 | 25.8% | 30.0% | 25.8% | 18.3% | 193.50 | 142.50-282.00 |
| Volleyball | 87 | 25.3% | 32.2% | 17.2% | 25.3% | 198.00 | 76.00-280.00 |
| Taekwondo | 83 | 30.1% | 15.7% | 24.1% | 30.1% | 178.00 | 76.00-300-00 |
| Chess | 59 | 18.6% | 23.7% | 30.5% | 27.1% | 166.00 | 84.00-248.00 |
| Hockey | 38 | 21.1% | 23.7% | 26.3% | 28.9% | 168.00 | 85.75-242.50 |
| Karate | 34 | 26.5% | 26.5% | 17.6% | 29.4% | 196.50 | 63.00-282.25 |
| Artistic skating | 33 | 21.2% | 36.4% | 12.1% | 30.3% | 211.00 | 85.50-265-50 |
| Skate-racing | 30 | 10.0% | 23.3% | 36.7% | 30.0% | 113.00 | 72.75-202.00 |
| Basque pelota | 25 | 32.0% | 32.0% | 16.0% | 20.0% | 229.00 | 115.50-322.50 |
| Multisport | 22 | 22.7% | 27.3% | 36.4% | 13.6% | 187.00 | 112.75-275.00 |
| Padel | 21 | 14.3% | 33.3% | 33.3% | 19.0% | 183.00 | 138.50-252.00 |
| Aerobic | 19 | 36.8% | 21.1% | 21.1% | 21.1% | 230.00 | 95.00-333.00 |
| Cycling | 18 | 16.7% | 16.7% | 16.7% | 50.0% | 98.50 | 56.50-251.75 |
| Judo | 16 | 25.0% | 31.3% | 31.3% | 12.5% | 218.00 | 149.50-275.00 |
| Tennis | 13 | 38.5% | 30.8% | 15.4% | 15.4% | 241.00 | 140.50-351.50 |
| Baseball | 12 | 33.3% | 8.3% | 33.3% | 25.0% | 144.00 | 96.50-337.00 |
| Synchronized sw | 9 | 22.2% | 33.3% | 44.4% |  | 226.00 | 127.00-283.00 |
| Rugby | 7 | 28.6% | 14.3% | 14.3% | 42.9% | 129.00 | 27.00-294.00 |
| Triathlon | 7 | 28.6% | 14.3% | 28.6% | 28.6% | 174.00 | 14.00-277.00 |
| Water polo | 7 | 14.3% | 42.9% | 42.9% |  | 186.00 | 178.00-271.00 |
| Climbing | 4 | 25.0% | 50.0% |  | 25.0% | 239.00 | 96.00-327.25 |
| Artistic Gym. | 4 | 25.0% | 50.0% | 25.0% |  | 242.50 | 166.00-279.25 |
| Trampolining | 3 | 33.3% | 33.3% | 33.3% |  | 250.00 |  |
| Canoeing | 1 | 100.0% |  |  |  |  |  |
| Table tennis | 1 |  | 100.0% |  |  |  |  |
| Archery | 1 |  |  |  | 100.0% |  |  |
| Total |  | 25.4% | 27.5% | 22.7% | 24.3% | 193.00 | 94.00-276.00 |
| Total (n) | 2502 | 636 | 689 | 568 | 609 |  |  |
| Gen pop (n) | 4833 | 1158 | 1257 | 1177 | 1241 |  |  |

Q: birth quarter; IQR: interquartile range (25^th^ and 75^th^ percentiles are shown); Gym: gymnastics; Trad: traditional; sw: swimming; Gen pop: general population
