# Supplementary material for: The relative age effect in young athletes: A countywide analysis of 9–14-year-old participants in all competitive sports
Source: PLoS One. 2021 Jul 16;16(7):e0254687. doi: 10.1371/journal.pone.0254687 (PMC8284647; doi:10.1371/journal.pone.0254687)
Supplement: S11 Table — (DOCX) [file pone.0254687.s011.docx]

**S11 Table.** Descriptive statistics of the birth dates of female 11-year-old participants and the general population.

|  | **Total (n)** | **Q1** | **Q2** | **Q3** | **Q4** | **Median** | **IQR** |
| --- | --- | --- | --- | --- | --- | --- | --- |
| Basketball | 711 | 26.9% | 27.1% | 23.2% | 22.8% | 196.00 | 103.00-279.00 |
| Rhythmic Gym | 261 | 24.9% | 26.8% | 22.6% | 25.7% | 194.00 | 88.00-274.50 |
| Football | 252 | 25.8% | 28.2% | 24.2% | 21.8% | 203.50 | 104.50-276.00 |
| Handball | 245 | 31.4% | 17.6% | 22.0% | 29.0% | 179.00 | 73.00-294.00 |
| Athletics | 225 | 22.7% | 29.8% | 24.4% | 23.1% | 198.00 | 99.00-266.50 |
| Swimming | 154 | 29.9% | 22.1% | 22.7% | 25.3% | 194.00 | 88.25-282.25 |
| Volleyball | 141 | 23.4% | 31.9% | 27.7% | 17.0% | 200.00 | 103.00-265.50 |
| Trad sport | 130 | 26.9% | 28.5% | 20.0% | 24.6% | 207.50 | 95.50-286.75 |
| Taekwondo | 77 | 26.0% | 23.4% | 22.1% | 28.6% | 182.00 | 69.50-277.00 |
| Chess | 56 | 28.6% | 16.1% | 28.6% | 26.8% | 173.50 | 90.25-280.50 |
| Artistic skating | 40 | 17.5% | 25.0% | 40.0% | 17.5% | 170.50 | 99.00-212.75 |
| Karate | 37 | 10.8% | 13.5% | 45.9% | 29.7% | 117.00 | 67.00-181.00 |
| Basque pelota | 37 | 29.7% | 35.1% | 10.8% | 24.3% | 228.00 | 87.00-298.50 |
| Hockey | 29 | 24.1% | 37.9% | 17.2% | 20.7% | 228.00 | 106.00-278.50 |
| Skate-racing | 29 | 17.2% | 37.9% | 17.2% | 27.6% | 208.00 | 69.50-263.00 |
| Synchronized sw | 28 | 21.4% | 28.6% | 35.7% | 14.3% | 174.50 | 110.50-270.00 |
| Aerobic | 24 | 12.5% | 20.8% | 37.5% | 29.2% | 149.00 | 85.75-216.25 |
| Judo | 23 | 21.7% | 21.7% | 34.8% | 21.7% | 146.00 | 115.00-266.00 |
| Baseball | 18 | 16.7% | 44.4% | 22.2% | 16.7% | 199.50 | 120.25-234.00 |
| Triathlon | 18 | 27.8% | 44.4% | 11.1% | 16.7% | 248.00 | 104.00-300.75 |
| Cycling | 15 | 6.7% | 40.0% | 26.7% | 26.7% | 166.00 | 90.00-252.00 |
| Tennis | 14 | 35.7% | 42.9% | 14.3% | 7.1% | 251.50 | 186.75-309.75 |
| Multisport | 14 | 21.4% | 7.1% | 28.6% | 42.9% | 146.00 | 80.75-236.25 |
| Padel | 13 | 23.1% | 15.4% | 38.5% | 23.1% | 180.00 | 92.00-250.00 |
| Canoeing | 8 | 12.5% | 25.0% | 50.0% | 12.5% | 158.50 | 111.50-272.50 |
| Water polo | 6 | 33.3% |  | 50.0% | 16.7% | 129.50 | 77.75-323.00 |
| Rowing | 5 | 40.0% | 20.0% | 40.0% |  | 190.00 | 128.50-321.00 |
| Rugby | 5 |  | 60.0% | 40.0% |  | 201.00 | 159.50-228.50 |
| Artistic Gym | 3 | 33.3% | 33.3% | 33.3% |  | 189.00 |  |
| Archery | 3 | 66.7% | 33.3% |  |  | 286.00 |  |
| Climbing | 2 |  | 50.0% | 50.0% |  | 187.50 |  |
| Table tennis | 2 | 50.0% |  |  | 50.0% | 196.5 |  |
| Total |  | 25.8% | 26.5% | 24.2% | 23.6% | 194.00 | 98-277.00 |
| Total (n) | 2625 | 676 | 695 | 635 | 619 |  |  |
| Gen pop (n) | 4730 | 1128 | 1215 | 1181 | 1206 |  |  |

Q: birth quarter; IQR: interquartile range (25^th^ and 75^th^ percentiles are shown); Gym: gymnastics; Trad: traditional; sw: swimming; Gen pop: general population
