# Supplementary material for: The relative age effect in young athletes: A countywide analysis of 9–14-year-old participants in all competitive sports
Source: PLoS One. 2021 Jul 16;16(7):e0254687. doi: 10.1371/journal.pone.0254687 (PMC8284647; doi:10.1371/journal.pone.0254687)
Supplement: S12 Table — (DOCX) [file pone.0254687.s012.docx]

**S12 Table.** Descriptive statistics of the birth dates of female 12-year-old participants and the general population.

|  | **Total (n)** | **Q1** | **Q2** | **Q3** | **Q4** | **Median** | **IQR** |
| --- | --- | --- | --- | --- | --- | --- | --- |
| Basketball (all) | 780 | 27.3% | 26.9% | 24.6% | 21.2% | 196.50 | 108.00-282.00 |
| Comp | 713 | 26.8% | 26.4% | 24.8% | 22.0% | 193.00 | 104.00-282.00 |
| Perf | 67 | 32.8% | 32.8% | 22.4% | 11.9% | 222.00 | 152.00-299.00 |
| Handball | 275 | 24.0% | 29.8% | 26.2% | 20.0% | 200.00 | 103.00-273.00 |
| Football | 248 | 25.8% | 27.4% | 22.6% | 24.2% | 199.00 | 95.50-278.75 |
| Rhythmic Gym. | 234 | 24.8% | 25.2% | 23.5% | 26.5% | 183.50 | 90.25-274.25 |
| Athletics | 192 | 24.5% | 29.7% | 24.5% | 21.4% | 207.50 | 103.50-274.00 |
| Volleyball | 174 | 25.3% | 27.6% | 24.7% | 22.4% | 217.00 | 108.00-279.00 |
| Swimming | 146 | 18.5% | 32.9% | 24.0% | 24.7% | 187.00 | 91.25-261.25 |
| Trad. Sport | 121 | 25.6% | 24.8% | 25.6% | 24.0% | 185.00 | 92.50-279.50 |
| Taekwondo | 77 | 28.6% | 29.9% | 26.0% | 15.6% | 211.00 | 136.50-288.00 |
| Karate | 51 | 25.5% | 23.5% | 31.4% | 19.6% | 178.00 | 115.00-282.00 |
| Hockey | 44 | 13.6% | 29.5% | 29.5% | 27.3% | 144.00 | 84.00-245.50 |
| Chess | 33 | 30.3% | 27.3% | 15.2% | 27.3% | 209.00 | 77.00-304.50 |
| Baseball | 27 | 14.8% | 18.5% | 25.9% | 40.7% | 130.00 | 59.00-226.00 |
| Basque pelota | 26 | 34.6% | 34.6% | 11.5% | 19.2% | 242.00 | 124.25-302.75 |
| Artistic skating | 20 | 20.0% | 25.0% | 25.0% | 30.0% | 139.50 | 44.00-249.00 |
| Skate-racing | 19 | 15.8% | 31.6% | 47.4% | 5.3% | 171.00 | 135.00-249.00 |
| Cycling | 17 | 5.9% | 41.2% | 29.4% | 23.5% | 169.00 | 91.00-256.50 |
| Padel | 16 | 25.0% | 25.0% | 25.0% | 25.0% | 189.50 | 80.50-294.75 |
| Triathlon | 16 | 37.5% | 25.0% | 12.5% | 25.0% | 233.00 | 86.50-311.75 |
| Tennis | 15 | 26.7% | 13.3% | 33.3% | 26.7% | 164.00 | 83.00-276.00 |
| Judo | 13 | 23.1% | 23.1% | 23.1% | 30.8% | 178.00 | 49.50-262.00 |
| Synchronized sw | 13 | 23.1% | 30.8% | 15.4% | 30.8% | 192.00 | 69.50-286.00 |
| Water polo | 13 | 15.4% | 15.4% | 53.8% | 15.4% | 154.00 | 109.00-204.50 |
| Aerobic | 12 | 25.0% | 16.7% | 33.3% | 25.0% | 130.00 | 89.00-279.25 |
| Artistic Gym. | 8 | 37.5% | 37.5% |  | 25.0% | 207.00 | 102.75-307.50 |
| Rowing | 8 | 37.5% | 25.0% | 12.5% | 25.0% | 248.00 | 65.75-356.50 |
| Rugby | 6 | 33.3% |  | 16.7% | 50.0% | 111.00 | 28.00-297.50 |
| Climbing | 5 | 20.0% | 20.0% | 40.0% | 20.0% | 182.00 | 105.00-273.00 |
| Archery | 4 |  |  | 75.0% | 25.0% | 132.00 | 82.25-170.50 |
| Canoeing | 3 | 33.3% |  |  | 66.7% | 77.00 |  |
| Table tennis | 3 | 66.7% |  |  | 33.3% | 323.00 |  |
| Trampolining | 2 | 50.0% |  |  | 50.0% | 140.00 |  |
| Multisport | 2 |  | 50.0% |  | 50.0% | 142.00 |  |
| Total |  | 25.2% | 27.4% | 24.7% | 22.7% | 193.00 | 101.00-275.00 |
| Total (n) | 2623 | 660 | 721 | 648 | 596 |  |  |
| Gen pop (n) | 4589 | 1092 | 1187 | 1152 | 1158 |  |  |

Q: birth quarter; IQR: interquartile range (25^th^ and 75^th^ percentiles are shown); Gym: gymnastics; Trad:

traditional; sw: swimming; Gen pop: general population
