# Supplementary material for: The relative age effect in young athletes: A countywide analysis of 9–14-year-old participants in all competitive sports
Source: PLoS One. 2021 Jul 16;16(7):e0254687. doi: 10.1371/journal.pone.0254687 (PMC8284647; doi:10.1371/journal.pone.0254687)
Supplement: S13 Table — (DOCX) [file pone.0254687.s013.docx]

**S13 Table.** Descriptive statistics of the birth dates of female 13-year-old participants and the general population.

|  | **Total (n)** | **Q1** | **Q2** | **Q3** | **Q4** | **Median** | **IQR** |
| --- | --- | --- | --- | --- | --- | --- | --- |
| Basketball (all) | 668 | 25.7% | 28.7% | 21.9% | 23.7% | 198.00 | 98.25-277.75 |
| Comp | 589 | 24.8% | 28.2% | 23.3% | 23.8% | 194.00 | 96.00-273.50 |
| Perf | 79 | 32.9% | 32.9% | 11.4% | 22.8% | 229.00 | 109.00-298.00 |
| Handball | 258 | 25.6% | 21.3% | 33.3% | 19.8% | 179.50 | 107.00-279.00 |
| Football | 232 | 27.2% | 28.4% | 20.7% | 23.7% | 200.00 | 96.00-287.75 |
| Rhythmic Gym. | 175 | 25.1% | 26.9% | 24.6% | 23.4% | 193.00 | 97.00-275.00 |
| Athletics | 174 | 28.7% | 31.0% | 20.7% | 19.5% | 223.50 | 113.75-289.50 |
| Volleyball | 93 | 29.0% | 21.5% | 29.0% | 20.4% | 201.00 | 99.00-291.00 |
| Taekwondo | 63 | 15.9% | 27.0% | 25.4% | 31.7% | 153.00 | 82.00-231.00 |
| Swimming | 59 | 33.9% | 13.6% | 22.0% | 30.5% | 177.00 | 59.00-293.00 |
| Aerobic | 24 | 8.3% | 33.3% | 29.2% | 29.2% | 167.50 | 70.75-239.50 |
| Basque pelota | 23 | 26.1% | 21.7% | 21.7% | 30.4% | 160.00 | 70.00-283.00 |
| Triathlon | 22 | 18.2% | 22.7% | 36.4% | 22.7% | 171.00 | 92.25-256.00 |
| Artistic skating | 21 | 28.6% | 28.6% | 28.6% | 14.3% | 217.00 | 139.50-288.50 |
| Karate | 20 | 15.0% | 35.0% | 20.0% | 30.0% | 189.50 | 84.25-244.00 |
| Trad. Sport | 18 | 11.1% | 44.4% | 22.2% | 22.2% | 197.00 | 100.00-229.00 |
| Chess | 15 | 13.3% | 26.7% | 26.7% | 33.3% | 151.00 | 41.00-251.00 |
| Hockey | 15 | 13.3% | 20.0% | 40.0% | 26.7% | 148.00 | 87.00-229.00 |
| Tennis | 15 |  | 26.7% | 40.0% | 33.3% | 133.00 | 69.00-190.00 |
| Judo | 14 | 28.6% | 7.1% | 28.6% | 35.7% | 150.00 | 58.75-298.00 |
| Padel | 14 | 14.3% | 21.4% | 28.6% | 35.7% | 143.00 | 44.25-237.00 |
| Skate-racing | 12 | 16.7% | 41.7% | 25.0% | 16.7% | 201.00 | 101.50-262.00 |
| Rugby | 12 | 50.0% | 16.7% | 25.0% | 8.3% | 259.50 | 148.25-311.50 |
| Rowing | 11 | 9.1% | 27.3% | 45.5% | 18.2% | 177.00 | 118.00-269.00 |
| Cycling | 9 | 44.4% | 33.3% |  | 22.2% | 273.00 | 133.00-332.50 |
| Synchronized sw | 8 | 62.5% | 12.5% | 12.5% | 12.5% | 291.50 | 138.75-301.50 |
| Canoeing | 6 | 16.7% | 33.3% | 16.7% | 33.3% | 155.50 | 72.00-233.50 |
| Water polo | 6 | 50.0% | 16.7% | 16.7% | 16.7% | 258.00 | 147.75-310.00 |
| Climbing | 4 |  | 50.0% | 50.0% |  | 181.00 | 127.75-231.25 |
| Artistic Gym. | 4 | 50.0% |  | 25.0% | 25.0% | 221.50 | 72.25-338.50 |
| Trampolining | 4 | 50.0% |  | 25.0% | 25.0% | 221.50 | 72.25-338.50 |
| Archery | 4 | 50.0% | 25.0% | 25.0% |  | 283.001 | 191.75-351.00 |
| Table tennis | 1 |  |  | 100.0% |  |  |  |
| Total |  | 25.6% | 26.6% | 24.6% | 23.2% | 192.00 | 98.00-277.50 |
| Total (n) | 2004 | 513 | 533 | 493 | 465 |  |  |
| Gen pop (n) | 4318 | 1051 | 1088 | 1090 | 1089 |  |  |

Q: birth quarter; IQR: Interquartile range (Percentile 25 and Percentile 75 are shown); Gym: gymnastics; trad:

traditional; sw: swimming; Gen pop: general population
