# Supplementary material for: The relative age effect in young athletes: A countywide analysis of 9–14-year-old participants in all competitive sports
Source: PLoS One. 2021 Jul 16;16(7):e0254687. doi: 10.1371/journal.pone.0254687 (PMC8284647; doi:10.1371/journal.pone.0254687)
Supplement: S14 Table — (DOCX) [file pone.0254687.s014.docx]

**S14 Table.** Descriptive statistics of the birth dates of female 14-year-old participants and the general population.

|  | **Total (n)** | **Q1** | **Q2** | **Q3** | **Q4** | **Median** | **IQR** |
| --- | --- | --- | --- | --- | --- | --- | --- |
| Basketball (all) | 504 | 28.0% | 26.2% | 23.2% | 22.6% | 200.00 | 99.00-284.75 |
| Comp | 404 | 26.7% | 27.7% | 22.5% | 23.0% | 198.50 | 98.25-279.75 |
| Perf | 100 | 33.3% | 19.8% | 26.0% | 20.8% | 209.50 | 102.00-308.75 |
| Aerobic | 24 | 33.3% | 12.5% | 33.3% | 20.8% | 165.00 | 94.50-295.00 |
| Football | 209 | 29.2% | 29.2% | 19.6% | 22.0% | 217.00 | 104.50-293.50 |
| Handball | 178 | 23.6% | 28.7% | 25.8% | 21.9% | 188.00 | 113.50-269.00 |
| Athletics | 167 | 27.5% | 26.9% | 24.6% | 21.0% | 200.00 | 107.00-281.00 |
| Volleyball | 162 | 27.8% | 21.6% | 22.8% | 27.8% | 182.00 | 84.75-286.00 |
| Rhythmic Gym. | 131 | 19.8% | 23.7% | 24.4% | 32.1% | 151.00 | 70.00-251.00 |
| Taekwondo | 57 | 21.1% | 22.8% | 22.8% | 33.3% | 149.00 | 44.50-248.00 |
| Swimming | 51 | 21.6% | 25.5% | 17.6% | 35.3% | 175.00 | 78.00-268.00 |
| Artistic skating | 29 | 6.9% | 24.1% | 31.0% | 37.9% | 93.00 | 55.00-229.00 |
| Hockey | 22 | 13.6% | 22.7% | 22.7% | 40.9% | 123.00 | 70.75-233.75 |
| Padel | 22 | 18.2% | 27.3% | 18.2% | 36.4% | 172.00 | 55.75-257.50 |
| Tennis | 22 | 22.7% | 40.9% | 9.1% | 27.3% | 214.00 | 78.50-277.00 |
| Karate | 21 | 19.0% | 23.8% | 33.3% | 23.8% | 143.00 | 90.00-259.50 |
| Trad. Sport | 18 | 16.7% | 27.8% | 38.9% | 16.7% | 176.00 | 120.25-262.50 |
| Triathlon | 15 | 26.7% | 20.0% | 20.0% | 33.3% | 152.00 | 79.00-281.00 |
| Skiing | 11 | 9.1% | 27.3% | 36.4% | 27.3% | 143.00 | 82.00-218.00 |
| Canoeing | 9 | 55.6% | 33.3% |  | 11.1% | 280.00 | 245.50-309.00 |
| Water polo | 9 | 22.2% |  | 22.2% | 55.6% | 73.00 | 37.00-237.50 |
| Basque pelota | 8 | 12.5% |  | 50.0% | 37.5% | 124.50 | 39.25-169.00 |
| Judo | 7 | 28.6% |  | 14.3% | 57.1% | 89.00 | 39.00-320.00 |
| Rugby | 7 | 28.6% | 14.3% | 28.6% | 28.6% | 126.00 | 43.00-304.00 |
| Cycling | 6 | 33.3% |  | 16.7% | 50.0% | 132.00 | 20.50-290.25 |
| Climbing | 5 | 20.0% | 60.0% | 20.0% |  | 259.00 | 175.00-316.50 |
| Rowing | 5 | 40.0% | 20.0% | 20.0% | 20.0% | 213.00 | 86.00-302.00 |
| Chess | 4 |  | 25.0% | 50.0% | 25.0% | 130.50 | 63.50-178.00 |
| Artistic Gym. | 3 |  |  | 33.3% | 66.7% | 52.00 |  |
| Synchronized sw | 3 | 33.3% | 66.7% |  |  | 231.00 |  |
| Archery | 3 |  |  | 66.7% | 33.3% | 99.00 |  |
| Table tennis | 2 |  | 50.0% | 50.0% |  | 141.00 |  |
| Trampolining | 1 |  |  |  | 100.0% |  |  |
| Total |  | 25.4% | 25.5% | 23.6% | 25.5% | 188.00 | 88.00-277.00 |
| Total (n) | 1715 | 436 | 439 | 403 | 437 |  |  |
| Gen pop (n) | 4265 | 1047 | 1128 | 1005 | 1085 |  |  |

Q: birth quarter; IQR: interquartile range (25^th^ and 75^th^ percentiles are shown); Gym: gymnastics; Trad: traditional; sw: swimming; Gen pop: general population
